# Accelerated first-order optimization under nonlinear constraints

**DOI:** 10.1007/s10107-025-02224-1

**Published:** 2025-04-21

**Authors:** Michael Muehlebach, Michael I. Jordan

**Affiliations:** 1 Learning and Dynamical Systems, Max Planck Institute for Intelligent Systems, Max-Planck-Ring 4, 72076 Tuebingen, Baden-Wuerttemberg Germany; 2 Department of Electrical Engineering and Computer Science, University of California, Berkeley, 387 Soda Hall, Berkeley, California 94720 USA

**Keywords:** Constrained optimization, Nonlinear programming, Gradient-based methods, Machine learning, 90-C30, 65-K05, 62-J07, 70-E99

## Abstract

We exploit analogies between first-order algorithms for constrained optimization and non-smooth dynamical systems to design a new class of accelerated first-order algorithms for constrained optimization. Unlike Frank–Wolfe or projected gradients, these algorithms avoid optimization over the entire feasible set at each iteration. We prove convergence to stationary points even in a nonconvex setting and we derive accelerated rates for the convex setting both in continuous time, as well as in discrete time. An important property of these algorithms is that constraints are expressed in terms of velocities instead of positions, which naturally leads to sparse, local and convex approximations of the feasible set (even if the feasible set is nonconvex). Thus, the complexity tends to grow mildly in the number of decision variables and in the number of constraints, which makes the algorithms suitable for machine learning applications. We apply our algorithms to a compressed sensing and a sparse regression problem, showing that we can treat nonconvex $\ell ^p$ constraints ($p<1$) efficiently, while recovering state-of-the-art performance for $p=1$.

## Introduction

Our work is concerned with developing first-order algorithms for nonlinear constrained optimization problems of the following form:

1 $$\begin{aligned} \min _{x\in \mathbb {R}^n} f(x), \quad \text {s.t.} \quad g(x)\ge 0, \end{aligned}$$

where the function $f:\mathbb {R}^n \rightarrow \mathbb {R}$ defines the objective, the function $g:\mathbb {R}^n \rightarrow \mathbb {R}^{n_\text {g}}$ the constraints, and where *n* and $n_\text {g}$ are positive integers. We assume that *f* and *g* are continuous, *f* is coercive, and that the feasible set $\{x\in \mathbb {R}^n~|~g(x)\ge 0\}$ is compact. The applications we have in mind include problems in statistics, machine learning, and control theory, where *n* and $n_\text {g}$ are typically on the order of $10^6$.

We develop a new class of accelerated first-order algorithms for computing stationary points of (1). These algorithms have four distinctive features: (i) they rely on local linear approximations of the feasible set, thereby avoiding projections or optimizations over the entire feasible set at every iteration, (ii) they are conceptually easy to understand and easy to implement, (iii) their iteration complexity for convex problems is dimension-independent,^1^ and (iv) in many important cases (even nonconvex ones) their per-iteration complexity scales roughly linearly in the number of decision variables and the number of constraints.

An important aspect of our work is to lift position constraints to a velocity level, which naturally results in a local linear approximation of the feasible set. These approximations come in two variants, each providing a different trade-off between the per-iteration complexity and the resulting convergence guarantees. More precisely, in our algorithms the forward increments, $(x_{k+1}-x_k)/T$, where $T>0$ is the step size, will be constrained to the set

2 $$\begin{aligned} V_{\alpha }(x):=\{v\in \mathbb {R}^n~|~\nabla g_i(x)^{\mathsf {\tiny {T}}}v + \alpha g_i(x) \ge 0, ~\forall i\in I \}, \end{aligned}$$

where *I* either has the form $I=[n_\text {g} ]:=\{1,2,\dots ,n_\text {g}\}$ or the form $I_x:=\{~i\in [n_\text {g}]~|~g_i(x)\le 0\}$. The former version includes every constraint at every iteration, while the latter version includes only constraints that are violated at the current iterate *x*. The analysis of algorithms is significantly more challenging when $I=I_x$ compared to $I=[n_\text {g}]$. In the following we will consider both variants, however, non-asymptotic linear rates in discrete time will only be derived for $I=[n_\text {g}]$. We conjecture that the same rates can be achieved asymptotically for $I=I_x$, which has been shown in the non-accelerated situation in earlier work, see [1]. Our convergence results span both continuous-time and discrete-time models and are summarized in Table 1 (see the corresponding theorems for the precise statements).

**Table 1:** The table summarizes the convergence results presented in the main text, where $\mu$ and $\kappa$ denote the strong convexity constant and condition number of *f*, $L_l$ is the smoothness constant of the Lagrangian $f(x)-{\lambda ^*}^{\mathsf {\tiny {T}}}g(x)$ with $\lambda ^*$ an optimal multiplier of (1), and $\kappa _l=L_l/\mu$. Moreover, smt stands for smooth, str for strongly, cvx for convex, cve for concave, and conv. to stat. for convergence to stationary points

Setting	Rate	Version	Objective	Constraints	$L_l$	Result
Discrete time	$\mathcal {O}(\sqrt{\kappa _l} \log (1/\varepsilon ))$	$I=[n_\text {g}]$	smt/str cvx	smt cve	kwn	Theorem 4
	$\tilde{\mathcal {O}}(1/\sqrt{\varepsilon })$	$I=[n_\text {g}]$	smt/str cvx	smt cve	–	Corollary 5
	$\tilde{\mathcal {O}}(1/\sqrt{\varepsilon })$	$I=[n_\text {g}]$	smt cvx	smt cve	kwn	Corollary 6
	conv. to stat	$I=I_x$	smt	smt	–	Theorem 3
Continuous time	$\mathcal {O}(\sqrt{\kappa }\log (1/\varepsilon ))$	$I=I_x$	smt/str cvx	smt cve	–	Theorem 2
	$\mathcal {O}(1/\sqrt{\varepsilon })$	$I=I_x$	smt cvx	smt cve	–	Theorem 2
	conv. to stat	$I=I_x$	smt	smt	–	Theorem 1

The column under $L_l$ describes whether a bound on the smoothness constant $L_l$ needs to be known (knw) and the tolerance is denoted by $\varepsilon$. In the special case where *g* is linear, $\kappa _l$ reduces to $\kappa$, and our algorithm recovers the rate of accelerated projected gradient descent, without requiring projections or optimizations over the entire feasible set

Our treatment builds on recent progress in using tools from continuous-time dynamical systems to analyze discrete-time algorithms in gradient-based optimization [2–15]. Much of this work aims at understanding accelerated first-order optimization methods, such as Nesterov’s algorithm, by exposing links between differential and symplectic geometry, dynamical systems, and mechanics. While in the absence of constraints these analogies result in *smooth* dynamical systems, the current article presents analogies between constrained optimization and *non-smooth* dynamical systems. Indeed, one of the closest point of contacts with existing literature is the notion of Moreau time-stepping in non-smooth mechanics [16]. The important feature of Moreau time-stepping, which also lies at the heart of our work, is that smooth and non-smooth motion are treated on equal footing, which is achieved by discretizing a certain kind of differential inclusion [see, e.g., 17, 18].

Our approach can also be interpreted through the lens of the projected gradient methodology and indeed it has certain similarities to inexact projected gradient methods, as proposed by Wang and Liu [19] and Birgin et al. [20]. While projected gradient approaches have been successfully applied in various machine learning problems [see, e.g., 21, 22], the Frank–Wolfe algorithm has also received considerable attention in recent years [23]. The appeal of Frank–Wolfe is further increased by the fact that it provides a unified framework for many first-order machine learning algorithms in constrained settings, including support vector machines, online estimation of mixtures of probability densities, and boosting [24]. Recent results extend the Frank–Wolfe algorithm to the stochastic setting [25, 26], or improve on its relatively slow convergence rate [27, 28].

In some cases constraints can be handled very efficiently with mirror descent, [29, Ch. 3], where a non-Euclidean metric is introduced that adapts gradient descent to the specific type of objective function or the specific type of constraints at hand [30]. Although mirror descent is based on projections onto the feasible set, the non-Euclidean metric can improve on problem-specific constants. An important example is the optimization of linear functions over the unit simplex, which has applications in online machine learning [31]. Another important class of methods arises from the alternating direction of multipliers, which can also be formulated with inertial dynamics, as recently shown by Attouch [32] and Attouch et al. [33]. In fact, [32, 33] proposes analogies to smooth dynamical systems for understanding the convergence properties and asymptotic behavior of algorithms. Our work follows a similar guiding principle, focusing on non-smooth dynamics that arise from set-valued operators.

Compared to projected gradients, mirror descent, and the Frank–Wolfe algorithm, our approach avoids optimizing over the entire feasible set at each iteration and instead relies on sparse, local and convex approximations. This article focuses on accelerated gradient descent, building on the recent results of Muehlebach and Jordan [1], which treats gradient descent. Including constraints in momentum-based algorithms is challenging: The presence of constraints requires a need for sudden and large changes in momentum (impacts) in order to avoid infeasible iterates. This requires us to not only characterize the smooth motion (if constraints are absent or the solution slides along the boundary of the feasible set), but also the non-smooth motion (if the solution suddenly hits the boundary of the feasible set).

Specific problems which have the potential to benefit from our approach include planning problems in reinforcement learning and/or optimal control [see, e.g., 34], optimizations over nonconvex matrix manifolds (such as the set of orthogonal matrices [see, e.g., 35]), distance geometry problems that arise in computational chemistry/NMR spectroscopy [36], optimal transport problems [37], or supervised learning tasks that involve nonlinear constraints (for example in an imitation learning framework [38], where nonlinear constraints arise from stability requirements on the closed-loop system). We will also demonstrate our approach on $\ell ^p$-regularized inverse problems that arise in compressed sensing and signal processing, where we are not only able to obtain state-of-the-art results for $p=1$, but can also seamlessly handle the regime $0<p<1$.

The article is structured in the following way: Sect. 2 summarizes earlier work of Muehlebach and Jordan [1], which covers gradient descent and sets the stage for discussing momentum-based algorithms in Sect. 3. A variety of convergence results that capture both discrete-time and continuous-time models are presented in Sect. 4; in particular, in the nonconvex regime we establish convergence to stationary points and we derive accelerated rates in the convex regime. Sect. 5 presents numerical experiments, which include nonconvex sparse regression and compressed sensing problems. The paper concludes with a short discussion in Sect. 6.

## Velocity constraints

The fundamental idea of this work is to express constraints in terms of the forward increment or velocity of our algorithms instead of constraining the iterates or positions directly. As we will see shortly, this naturally leads to local, sparse and convex approximations of the feasible set. Our treatment builds upon Muehlebach and Jordan [1], which focused on gradient descent and gradient flow, whereas this work focuses on accelerated gradient algorithms. The section briefly summarizes the results from [1] in order to set the stage for deriving accelerated algorithms in Sect. 3 and quantify their convergence rates in Sect. 4.

We model an optimization algorithm as a continuous-time or discrete-time dynamical system, whose equilibria correspond to the stationary points of (1). In continuous time, the configuration of the system will be denoted by a function $x: [0,\infty ) \rightarrow \mathbb {R}^n$, which is assumed to be absolutely continuous. A fundamental observation, lying at the heart of the current research, is that the constraint $x(t) \in C$, for all $t\ge 0$, is equivalent to the constraint $\dot{x}(t)^+ \in T_C(x(t))$, for all $t\ge 0$, $x(0) \in C$, where $T_C(x(t))$ denotes the tangent cone (in the sense of Clarke) of the set *C* at $x(t) \in \mathbb {R}^n$, and $\dot{x}(t)^+$ denotes the forward velocity: $\dot{x}(t)^+:= \lim _{\textrm{d}t \downarrow 0} (x(t+\textrm{d}t )-x(t))/\textrm{d}t$. The tangent cone $T_C(x)$ is defined as the set of all vectors *v* such that $(x_k-x)/t_k \rightarrow v$ for two sequences $x_k\in C$ and $t_k\ge 0$ with $x_k\rightarrow x$, $t_k \rightarrow 0$. Provided that a constraint qualification holds (for example Mangasarian-Fromovitz or Abadie constraint qualification), the tangent cone can be expressed as

$$\begin{aligned} T_C(x)=\{ v\in \mathbb {R}^n ~|~ \nabla g_i(x)^{\mathsf {\tiny {T}}}v \ge 0, ~~\forall i \in I_x \}, \end{aligned}$$

where $I_x=\{i\in [n_\text {g}]~|~g_i(x)\le 0\}$ denotes the set of active inequality constraints at *x*.

We therefore conclude that the constraint $x(t)\in C$, which constrains the position *x*, is equivalent to a constraint on the forward velocity $\dot{x}^+$. We note that the velocity $\dot{x}$ is allowed to be discontinuous and need not exist for every $t\ge 0$.^2^ For example, if the trajectory *x* reaches the boundary of the feasible set, an instantaneous jump of the velocity might be required to ensure that *x* remains in *C*.

In discrete time, however, this equivalence between position and velocity constraints no longer holds, since $T_C(x)$ is only a first-order approximation of the feasible set. Thus, implementing $(x_{k+1}-x_k)/T \in T_C(x_k)$ may lead to infeasible iterates. Muehlebach and Jordan [1] therefore suggest to introduce the velocity constraint $V_\alpha (x)$, see (2) with $I=I_x$, which includes the restitution coefficient $\alpha > 0$. The following remarks motivate (2) and are important for understanding the accelerated algorithms presented subsequently:

(i) For $x\in C$, the set $V_\alpha (x)$ reduces to the tangent cone $T_C(x)$ (assuming constraint qualification).
(ii) For a fixed $x\in \mathbb {R}^n$, $V_\alpha (x)$ is a convex polyhedral set involving only the active constraints $I_x$. The set $V_\alpha (x)$ therefore amounts to a sparse and linear approximation of the feasible set *C*, even if *C* is nonconvex.
(iii) In continuous time, the constraint $\dot{x}(t)^+\in V_\alpha (x(t))$ for all $t\ge 0$ implies
  3 $$\begin{aligned} g_i(x(t))&\ge \min \{g_i(x(0)) e^{-\alpha t}, 0\}, \end{aligned}$$
  for all $t\ge 0$ and all $i\in \{1,\dots ,n_\text {g}\}$, which can be verified with Grönwall’s inequality. This means that potential constraint violations decrease at rate $\alpha$.

### Remark 1

Nonemptiness of $V_\alpha (x)$: If *C* is convex, $V_\alpha (x)$ is guaranteed to be nonempty for all $x\in \mathbb {R}^n$. If *C* is nonconvex, nonemptiness of $V_\alpha (x)$ for all *x* in a neighborhood of *C* is guaranteed if the Mangasarian-Fromovitz constraint qualification holds for all $x\in C$. We note that the Mangasarian-Fromovitz constraint qualification is generic in the following sense: Provided that *g* is semi-algebraic (these cases include all the usual functions used in optimization) there exists $\epsilon _0\in \mathbb {R}^m, \epsilon _0> 0$, such that the set $C_\epsilon :=\{x\in \mathbb {R}^n~|~g(x)\ge -\epsilon \}$ satisfies the Mangasarian-Fromovitz constraint qualification for all $x\in C_\epsilon$ and for all $\epsilon \in (0,\epsilon _0)$; see Bolte et al. [39].

The continuous-time gradient flow dynamics that were studied in Muehlebach and Jordan [1] arise from the following equation:

4 $$\begin{aligned} \dot{x}(t)^+ +\nabla f(x(t))=R(t), \quad -R(t)\in N_{V_\alpha (x(t))}(\dot{x}(t)^+), \end{aligned}$$

for all $t\ge 0$, where $N_{V_\alpha (x(t))}(\dot{x}(t)^+)$ denotes the normal cone of the set $V_\alpha (x(t))$ at $\dot{x}(t)^+$. Thus, the variable *R*(*t*) can be regarded as a constraint force that imposes the constraint $\dot{x}(t)^+\in V_\alpha (x(t))$. In discrete time, it suffices to replace $\dot{x}(t)^+$ by $(x_{k+1}-x_k)/T$ and *x*(*t*) by $x_k$ in order to obtain the corresponding constrained gradient-descent dynamics. These can be expressed as (see [1] for details)

5 $$\begin{aligned} x_{k+1} = x_k + T {\mathop {\textrm{argmin}}\limits _{v\in V_\alpha (x_k)}} |v+\nabla f(x_k)|^2, \end{aligned}$$

where $T>0$ is the step size and can be interpreted as a modified projected gradient scheme, where projections over the entire feasible set *C* are replaced with optimizations over the sparse and convex approximation $V_\alpha (x_k)$.

The results from Muehlebach and Jordan [1] establish convergence of (4) and (5). In continuous time, it was shown that even when *f* and *C* are nonconvex, the trajectories of (4) converge to the set of stationary points. Moreover, if *f* is strongly convex with strong convexity constant $\mu$ and $\alpha$ is set to $2\mu$, the trajectories converge from any initial condition to the minimizer of (1) at a linear rate, which scales with $1/\kappa$, where $\kappa$ is the condition number of *f*. A similar, albeit asymptotic, convergence rate was found in discrete time for an adequate choice of step size. However, a non-asymptotic linear convergence result was missing in discrete time; the asymptotic result required a relatively complex analysis of the algorithm’s dynamics. For completeness, we therefore provide a non-asymptotic convergence result in Appendix A (see Theorem 7).

The results from Theorem 7 can be compared to a type of composite optimization [see, e.g., 18, BertsekasComp,PaquetteComp,FletcherComp,BolteComp,NesterovComp,NicolasComp], which can also be applied to problems of the type (1) resulting in updates similar to (5). The resulting convergence rates known in the literature for the convex setting are similar to Theorem 7, see, e.g., [43, Thm. 2], [44, Thm. 5], [45],^3^ with the important difference that [43–45] do not require knowledge of the constant $L_l$. However, the current article highlights that much faster and accelerated rates can be achieved in both the smooth and strongly convex case, as well as in the smooth convex case by adding momentum.

To summarize, (4) and (5) implement gradient-flow and gradient-descent dynamics that can handle constraints and converge linearly with the typical $1/\kappa$ (continuous time) and $1/\kappa _l$-rate (discrete time) if the objective function *f* is smooth and strongly convex, where $\kappa _l=L_l/\mu$ and $L_l$ relates to the smoothness constant of the Lagrangian.^4^ The set $V_\alpha (x)$ can be seen as a velocity constraint and provides a natural generalization of the tangent cone. It also reduces the computational cost for each iteration, since projections on the entire feasible set are avoided. In the next section, we generalize these ideas to algorithms that have momentum. This will naturally lead to accelerated algorithms that converge linearly at a rate of $1/\sqrt{\kappa _l}$ (if *f* is smooth and strongly convex) or at the sublinear rate $1/t^2$ (if *f* is smooth and convex), which is a significant speedup. We will also derive convergence if *f* and *C* are nonconvex.

## Accelerated gradient flow

We begin our presentation with a derivation in continuous time. The corresponding discrete-time algorithms will be stated subsequently. A natural starting point is the work of authors such as Polyak [46], Su et al. [2], and Muehlebach and Jordan [8], who argued that in the *unconstrained* case, accelerated optimization algorithms can be viewed as dynamical systems described by second-order differential equations. A canonical example is the following:

6 $$\begin{aligned} \dot{u}(t)+2 \delta u(t) +\nabla f(x(t)+\beta u(t))=0, \end{aligned}$$

where we use the variable $u(t)=\dot{x}(t)$ to denote the velocity (or momentum), and where $\delta \ge 0$ and $\beta \ge 0$ are damping parameters.^5^

In the presence of constraints, *u*(*t*) is allowed to be discontinuous, which is in sharp contrast to (6). For example, if the trajectory *x*(*t*) approaches the boundary of the feasible set, an instantaneous jump in *u*(*t*) might be required to ensure that *x*(*t*) remains feasible. Thus, compared to (4), where the state *x*(*t*) is absolutely continuous, we are now in a position where we allow for the state (*x*(*t*), *u*(*t*)) (which includes the velocity *u*) to be discontinuous. This means that in addition to a differential equation of the type (4), which characterizes the smooth motion, we also prescribe how the discontinuities in *u* can arise. If we regard (*x*(*t*), *u*(*t*)) as the position and velocity of a mechanical system, discontinuities in *u* have a mechanical meaning as impacts, which are described by a corresponding impact law. The mathematical formalism, which enables discontinuities in *u*, is summarized next.

We regard the state $z:=(x,u)$ to be the result of an integration process:

$$\begin{aligned} z(t)=z(t_0) + \int _{t_0}^{t} \textrm{d}z, \quad \forall t\ge t_0. \end{aligned}$$

However, instead of the usual Lebesgue density $\textrm{d}z=\dot{z}(t)\textrm{d}t$, $\textrm{d}z$ now represents a differential measure [47], and admits both a density with respect to the Lebesgue measure (denoted by $\textrm{d}t$), as well as a density with respect to an atomic measure (denoted by $\textrm{d}\eta$). As is common in non-smooth mechanics, we assume that *z*(*t*) is of locally bounded variation and does not contain any singular terms. This means that *z*(*t*) can be decomposed as an absolutely continuous function and a piecewise constant step function [47]. At every time *t*, *z*(*t*) has well-defined left and right limits, $z(t)^-$ and $z(t)^+$, even though *z*(*t*) might not exist or might not be of interest. We can express the differential measure $\textrm{d}z$ as $\textrm{d}z=\dot{z}(t)\textrm{d}t + (z(t)^+-z(t)^-) \textrm{d}\eta ,$ and the integration over an interval $[t_0,t]$, which contains the time instants $t_{\text {d}i}$, $i=1,2,\dots$, where *z*(*t*) is discontinuous, yields

$$\begin{aligned} z(t)^+=z(t_0)^- + \int _{t_0}^{t} \dot{z}(t) \textrm{d}t + \sum _{i\ge 1} z(t_{\text {d}i})^+-z(t_{\text {d}i})^-. \end{aligned}$$

**Fig. 1:**
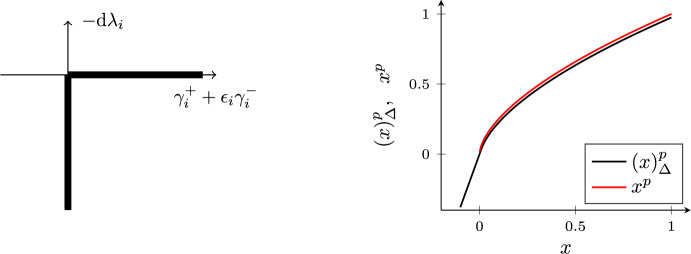
The left panel shows the normal cone inclusion $\gamma _i^+ +\epsilon \gamma _i^-\in N_{\mathbb {R}_{\le 0}}(-\textrm{d}\lambda _i)$, which is equivalent to the complementarity condition $\textrm{d}\lambda _i\ge 0$, $\gamma _i^+ + \epsilon \gamma _i^- \ge 0$, $\textrm{d}\lambda _i (\gamma _i^+ + \epsilon \gamma _i^-)=0$. The right panel shows the approximation $(x)^p_\Delta$ of $x^p$ for $\Delta =0.01$ and $p=0.6$. There is an excellent agreement between the approximation and $x^p$ even though $\Delta$ is comparably large. In the numerical experiments, see Sect. 5, $\Delta$ is set to $10^{-6}$

As a consequence of allowing the state to be discontinuous, we need to delineate both the density $\dot{z}(t)$ with respect to the Lebesgue measure $\textrm{d}t$ (which describes the smooth part of the motion) as well as the density $z(t)^+-z(t)^-$ (which describes the non-smooth motion) for fully determining the state trajectory *z*(*t*). By analogy to non-smooth mechanics [see, e.g., 18], this can be achieved with the following measure-differential inclusion:

7 $$\begin{aligned} \textrm{d}u + 2 \delta u \textrm{d}t + \nabla f(x+\beta u)\textrm{d}t = \sum _{i\in I_x} \nabla g_i(x) \textrm{d}\lambda _i,\quad \gamma _i^+ + \epsilon \gamma _i^- \in N_{\mathbb {R}_{\le 0}}(-\textrm{d}\lambda _i), \quad i\in I_x, \end{aligned}$$

where $\epsilon \in [0,1)$ is a constant, $\gamma _i$ is the velocity associated with the *i*th constraint and is defined as

$$\begin{aligned} \gamma _i(x,u):=\nabla g_i(x)^{\mathsf {\tiny {T}}}u + \alpha g_i(x), \end{aligned}$$

and where we have omitted the dependence on *t* (as we will do frequently in the subsequent presentation). We note that the set $I_x$ (or $I_{x(t)}$ in full notation) is time-dependent. The normal cone inclusion in (7) is illustrated with Fig. 1 and will be further discussed below. The constant $\epsilon$ has the interpretation of a restitution coefficient, whereby $\epsilon =0$ leads to inelastic collisions, and $\epsilon =1$ yields elastic collisions. We use the following solution concept for (7):

### [Style1 Style3 Style3]Definition 1

(*Solution of a measure differential inclusion, see* [47, Def. 4.7]) A solution $z(t)=\varphi (t,t_0,z_0)$ of the measure differential inclusion (7) with initial condition $z(t_0)^-=z_0$ is a function $z:\mathbb {R} \rightarrow \mathbb {R}^{2n}$, being of locally bounded variation, which fulfills (7) and $\textrm{d}x=u(t) \textrm{d}t$ for all $t\ge t_0$ and which is not defined at its discontinuity points.

Measure-differential inclusions are common in non-smooth mechanics. However, establishing existence of solutions is challenging, and solutions are rarely unique. Exceptions arise in situations where the right-hand side of the differential inclusion is monotone, which is, however, too restrictive for our purposes. We refer the reader for existence results to the literature, see, for example [48, 49] and references therein.

We note that if *x* is in the interior of the feasible set, $I_x$ is empty, and therefore (7) reduces to (6). This means that (7) generalizes (6) from the unconstrained case to the constrained case by including the constraint $\gamma _i^+ + \epsilon \gamma _i^- \in N_{\mathbb {R}_{\le 0}} (-\textrm{d}\lambda _i)$, which, as we will discuss below, describes the discontinuities of *u* via Newton’s impact law and imposes the velocity constraint $u(t)\in V_\alpha (x(t))$, whenever *u*(*t*) exists.

It is important to note that (7) is understood in the sense of integration: For any compact time interval $[t_0,t_1]$, (7) defines the difference $u(t_1)^+-u(t_0)^-$, which is obtained by integrating $\textrm{d}u$ from $t_0$ to $t_1$; similarly, the difference $x(t_1)^+-x(t_0)^-$ is obtained by integrating $u(t) \textrm{d}t$. This means that (7) has a very natural discretization, which will be discussed in the next paragraph. The second and the third paragraphs describe the interpretation of (7) in terms of its smooth and non-smooth components. Formal convergence results in continuous and discrete time will be derived in Sect. 4.

***Discretization of*** (7):

The measure-differential inclusion (7) lends itself to the following discretization: $\textrm{d}u=u_{k+1}-u_k$, $\textrm{d}t = T_k$, $\textrm{d}\lambda _i=\Lambda _{ki}$, $\gamma _i^+=\gamma _i(x_{k},u_{k+1})$, $\gamma _i^-=\min \{0,\gamma _i(x_k,u_k)\}$, where $T_k>0$ is the step size.^6^ This yields

$$\begin{aligned} u_{k+1}-u_k + {2}\delta u_k T_k + \nabla f(x_k + \beta u_k) T_k&= \sum _{i\in I_{x_k}} \nabla g_i(x_k) \Lambda _{ki},\\ \gamma _i(x_k,u_{k+1}) + \epsilon \min \{0,\gamma _i(x_k, u_k)\}&\in N_{\mathbb {R}_{\le 0}}(-\Lambda _{ki}), \quad i\in I_{x_k}. \end{aligned}$$

We use the newly computed momentum for updating the position $x_k$: $x_{k+1}=x_k + T_k u_{k+1}$, which is motivated by analogy to unconstrained optimization. (This discretization scheme is found to be superior compared to the standard Euler method [10].) The resulting update for $u_{k+1}$ can be interpreted as a stationarity condition for $u_{k+1}$, and as a result, the proposed algorithm can be summarized as follows: 
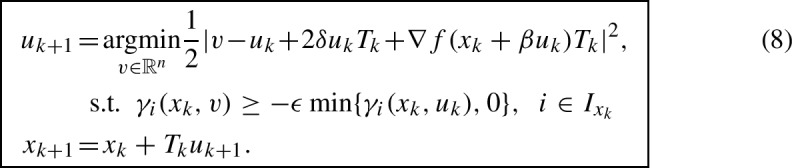
 Remark 1 applies here in the same way: If *C* is convex and $\epsilon =0$, the feasible set in (8) is guaranteed to be nonempty, which means that $u_{k+1}$ is well defined (existence and uniqueness). If *C* is nonconvex or $\epsilon > 0$, nonemptiness of the feasible set is guaranteed if constraint qualifications are satisfied (for example Mangasarian-Fromovitz). These constraint qualifications are generic, as discussed in Remark 1, which ensures that $u_{k+1}$ is well defined as long as $x_k$ stays in a neighborhood of the feasible set. As will be shown with our convergence analysis (see Sect. 4), we can indeed ensure that $x_k$ remains in a neighborhood of *C*; the size of the neighborhood can be controlled by the value of $T_k$. The pseudo-code of the full algorithm is listed in Appendix B.

The following remarks are important:

(i) The update (8) has the interpretation of choosing $u_{k+1}$ to be as close as possible to the update in the unconstrained case subject to the velocity constraint $\gamma _i(x_k,u_{k+1}) \ge -\epsilon \min \{\gamma _i(x_k,u_k),0\}$. As a result, in case $I_{x_k}$ is empty, (8) reduces to a standard momentum-based method; if $\beta =0$ we obtain the heavy-ball algorithm, if $\beta \ne 0$ we obtain Nesterov’s method.
(ii) The update (8) includes only the constraints $I_{x_k}$ which are active at iteration *k*. The constraint on *v* in (8) is guaranteed to be convex, even if the underlying feasible set is nonconvex. The constraints in (8) yield therefore a sparse, local and convex approximation of the feasible set. Instead of performing optimizations on the position level as is common with projected gradients or the Frank–Wolfe method, (8) suggests to constrain the velocities $u_k$, $k=1,2,\dots$.

We now proceed to give an interpretation and explanation of the continuous-time dynamics (7).

***Smooth motion:***

If *u*(*t*) happens to be absolutely continuous in the interval $(t_0,t_1)$, its differential measure reduces to $\dot{u}(t)\textrm{d}t$. Similarly, the multipliers $\textrm{d}\lambda _i$ have only a density with respect to the Lebesgue measure $\textrm{d}t$, which we denote by $\lambda _i(t)$, i.e., $\textrm{d}\lambda _i=\lambda _i(t) \textrm{d}t$. As a result, (7) reduces to

9 $$\begin{aligned} \dot{u} + 2\delta u + \nabla f(x+\beta u) = \sum _{i\in I_{x}} \nabla g_i(x)\lambda _i, \end{aligned}$$

for all $t\in (t_0,t_1)$ (a.e.). Furthermore, absolute continuity of *u*(*t*) implies absolute continuity of $\gamma _i$, i.e., $\gamma _i^+=\gamma _i^-=\gamma _i$. In the limit $\textrm{d}t \downarrow 0$, the inclusion in (7) therefore reduces to

$$\begin{aligned} (1+\epsilon ) \gamma _i \in N_{\mathbb {R}_{\le 0}} (-\lambda _i) \quad \Leftrightarrow \quad \gamma _i \in N_{\mathbb {R}_{\le 0}} (-\lambda _i), \end{aligned}$$

for all $i\in I_{x(t)}$ and for all $t\in (t_0,t_1)$ (a.e.). ($N_{\mathbb {R}_{\le 0}}$ is a cone; we can therefore divide by $1+\epsilon >0$.) The normal cone inclusion prescribing the relationship between $\gamma _i$ and $\lambda _i$ is similar to Fig. 1.

From a physics perspective the normal cone inclusion $\gamma _i \in N_{\mathbb {R}\le 0} (-\lambda _i)$ represents a force law, which by conic duality can also be expressed as (see again Fig. 1)

$$\begin{aligned} -\lambda _i \in N_{\mathbb {R} \ge 0} (\gamma _i). \end{aligned}$$

The sum $\nabla g_i(x(t)) \lambda _i$ over $i\in I_x$ on the right-hand side of (9) therefore has a physical interpretation as a constraint force:

$$\begin{aligned} -R=-\sum _{i\in I_x} \nabla g_i(x) \lambda _i \in N_{V_\alpha (x)} (u), \end{aligned}$$

which imposes the velocity constraint $u(t)\in V_\alpha (x(t))$ for all $t\in (t_0,t_1)$ (a.e.). By virtue of Grönwall’s inequality this ensures that constraint violations decrease linearly at rate $\alpha$, as highlighted in (3).

We therefore conclude that in case of smooth motion, the measure-differential inclusion (7) generalizes the differential equation (6) from the unconstrained case to the constrained case, where the additional constraint force *R*(*t*) imposes the velocity constraint $u(t)\in V_\alpha (x(t))$ (for almost all *t*). The introduction of the force *R*(*t*) is analogous to (4).

Since the motion is smooth for almost every *t*, the normal cone inclusion in (7) guarantees the satisfaction of the velocity constraint $u(t)\in V_{\alpha }(x(t))$ (or equivalently, $\gamma _i(x(t),u(t))\ge 0$ for all $i\in I_{x(t)}$) for all $t\ge 0$ (a.e.). However, when a new constraint arises at time $t_0$, there might be a situation where $\gamma _i^-(x(t_0),u(t_0))<0$. In such a case an impact will be required to ensure that $\gamma _i^+(x(t_0),u(t_0))\ge 0$. This is the subject of the next paragraph.

***Non-smooth motion:***

In order to derive the non-smooth motion we integrate (7) over a time instant $\{t\}$, where *u*(*t*) is discontinuous; that is, $u(t)^- \ne u(t)^+$. Due to the fact that the singleton $\{t\}$ has zero Lebesgue measure, we are left with the atomic parts, leading to $\textrm{d}u=(u(t)^+-u(t)^-)\textrm{d}\eta$, $\textrm{d}\lambda _i:=\Lambda _i \textrm{d}\eta$,

10 $$\begin{aligned} u(t)^+-u(t)^-=\sum _{i\in I_{x(t)}} \nabla g_i(x(t)) \Lambda _i,\quad \gamma _i^+ + \epsilon \gamma _i^- \in N_{\mathbb {R}\le 0}(-\Lambda _i), \quad i\in I_{x(t)}. \nonumber \\ \end{aligned}$$

The normal cone inclusion should be interpreted as a generalization of Newton’s impact law. For $\Lambda _i>0$, it implies $\gamma _i^+ + \epsilon \gamma _i^-=0$, meaning that the velocity associated to constraint *i* after impact, $\gamma _i^+$, is $-\epsilon \gamma _i^-$, where $\gamma _i^-$ is the velocity associated to constraint *i* before impact. From the discussion of the smooth motion it follows $\gamma _i(x(t_0),u(t_0))^-$ at time $t_0$ can only be negative if the constraint *i* becomes active at time $t_0$; that is, $i\not \in I_{x(t)}$ for $t<t_0$ and $i\in I_{x(t)}$ for $t=t_0$. This necessitates a discontinuity in *u* at time $t_0$, which according to the above normal cone inclusion comes in two variants: (i) $\Lambda _i>0$, which implies $\gamma _i^+=-\epsilon \gamma _i^-$ and (ii) $\Lambda _i=0$, which implies $\gamma _i^+\ge -\epsilon \gamma _i^-$. In variant (i), the impulsive force $\Lambda _i$ contributes the component $\Lambda _i \nabla g_i(x(t))$ (normal to constraint *i*) to the velocity jump $u(t_0)^+-u(t_0)^-$, whereas in variant (ii), there is no such contribution. Both variants ensure $\gamma _i(x(t_0),u(t_0))^+\ge -\epsilon \gamma _i(x(t_0),u(t_0))^-\ge 0$.

The characterization of the non-smooth motion according to (10) can be interpreted as a stationarity condition for $u(t)^+$, which yields

11 $$\begin{aligned} \begin{array}{c} u(t)^+=\mathop {\textrm{argmin}}_{v\in \mathbb {R}^n} \frac{1}{2} |v-u(t)^-|^2\quad \text {s.t.}~~ \gamma _i(x(t),v) \ge -\epsilon \gamma _i(x(t),u(t)^-),~~\forall i\in I_{x(t)}. \end{array}\end{aligned}$$

The minimization in (11) has the following meaning: for each $u(t)^-$ there is a unique $u(t)^+$, which is chosen to be as close as possible to $u(t)^-$ subject to Newton’s impact law $\gamma _i^+\ge -\epsilon \gamma _i^-$ for all $i\in I_{x(t)}$.

***Equilibria of*** (7):

The equilibria of (7) are obtained from $x(t)\equiv x_0$, $\textrm{d}\lambda _i \equiv \lambda _{0i} \textrm{d}t$, $u(t)\equiv 0$, $\textrm{d}u\equiv 0$, where $x_0\in \mathbb {R}^n$ and the multipliers $\lambda _{0i}\ge 0$, $i\in I_{x_0}$ are constant. As a result, (7) reduces to

$$\begin{aligned}\begin{array}{c} -\nabla f(x_0)+{\sum }_{i\in I_{x_0}}\nabla g_i(x_0) \lambda _{i0}=0, ~~ (1+\epsilon ) \alpha g_i(x_0) \in N_{\mathbb {R}_{\le 0}}(-\lambda _{i0}), ~~i\in I_{x_0}. \end{array}\end{aligned}$$

The normal cone inclusion can be simplified by dividing by $\alpha (1+\epsilon )>0$ (the normal cone is a cone), which implies that $g_i(x_0)$ and $\lambda _{i0}$ satisfy the complementarity conditions

$$\begin{aligned} g_i(x_0)\ge 0, \quad \lambda _{i0} \ge 0, \quad \lambda _{i0} g_i(x_0)=0, \quad \forall i\in I_{x_0}. \end{aligned}$$

Hence, the equilibria of (7) satisfy the Karush-Kuhn-Tucker conditions of (1), which means that the stationary points of (1) are indeed equilibria.

## Convergence analysis

The following section discusses the convergence of trajectories of (7) and (8), and characterizes the rate of convergence. We start by summarizing the continuous-time results. Without loss of generality we assume that *f* is normalized such that the Lipschitz constant of the gradient is unity.

### Theorem 1

Let (*x*(*t*), *u*(*t*)) be a trajectory satisfying (7) with $x(0)\in C$. Let *f* be 1-smooth, let *g* satisfy the Mangasarian-Fromovitz constraint qualification, and let either *f* be convex or $2\delta - \beta > 0$. Then, *x*(*t*) converges to the set of stationary points, while *u*(*t*) converges to zero. Moreover, each isolated local minimum corresponds to an asymptotically stable equilibrium in the sense of Lyapunov.

### Proof

From the analysis of the smooth motion in Sect. 3, we infer that $\gamma _i(x(t),u(t))\ge 0$ for all *t* such that $i\in I_{x(t)}$ (a.e.). By Grönwall’s inequality, this implies $g(x(t))\ge 0$ for all $t\ge 0$ and therefore $x(t) \in C$ for all $t\in [0,\infty )$.

We consider the following Lyapunov function

12 $$\begin{aligned} {V(t)}=\frac{1}{2} |u(t)|^2 + f(x(t)), \end{aligned}$$

which is bounded below by assumption. We investigate how *V* evolves along the trajectories of (7). The differential measure corresponding to *V* can be expressed as [47]:

$$\begin{aligned} \textrm{d}V&= \frac{1}{2} (u^+ + u^-)^{\mathsf {\tiny {T}}}\textrm{d}u + \nabla f(x)^{\mathsf {\tiny {T}}}u ~\textrm{d}t \\&= -2 \delta |u|^2 ~\textrm{d}t - (\nabla f(x+\beta u)-\nabla f(x))^{\mathsf {\tiny {T}}}u ~\textrm{d}t \\&\quad +\sum _{i\in I_{x}} \left( \frac{1}{2} (\gamma _i^+ + \gamma _i^-) - \alpha {g_i(x)}\right) \textrm{d}\lambda _i. \end{aligned}$$

The second line follows from replacing $\textrm{d}u$ with (7), using the fact that the Lebesgue measure captures only the smooth motion, which means, for example, $(u^+)^{\mathsf {\tiny {T}}}u~\textrm{d}t =(u^-)^{\mathsf {\tiny {T}}}u~\textrm{d}t =|u|^2 \textrm{d}t$, and adding and subtracting $\alpha g_i\textrm{d}\lambda _i$. From the assumption $2\delta - \beta >0$ (or alternatively by convexity of *f*) we can upper bound $\textrm{d}V$ by

$$\begin{aligned} \textrm{d}V \le -c_2 |u|^2 \textrm{d}t + \sum _{i\in I_x} \left( \frac{1}{2}(\gamma _i^+ +\gamma _i^-) - \alpha {g_i(x)} \right) \textrm{d}\lambda _i, \end{aligned}$$

where $c_2>0$ is constant. The summand in the second part of the expression can be rewritten as

$$\begin{aligned} -\alpha {g_i(x)} \textrm{d}\lambda _i + \frac{1}{2} (\gamma _i^+ + \epsilon \gamma _i^-) \textrm{d}\lambda _i + \frac{1-\epsilon }{2} (\gamma _i^-) \textrm{d}\lambda _i. \end{aligned}$$

The fact that $g_i(x(t))=0$ for all $i\in I_{x(t)}$ (*x*(*t*) remains feasible) implies that the first term vanishes. The second term vanishes due to the complementarity condition in (7). The third term is guaranteed to be non-positive, since, on the one hand, $\textrm{d}\lambda _i\ge 0$ (see (7)), and on the other hand, $\gamma _i^-\le 0$ in case of impact, and $\gamma _i^-~\textrm{d}\lambda _i=\gamma _i \textrm{d}\lambda _i=0$ in case of smooth motion (see again (7)). We therefore conclude that

13 $$\begin{aligned} \textrm{d}V \le -c_2 |u|^2 \textrm{d}t , \end{aligned}$$

which means that *V*(*x*(*t*), *u*(*t*)) is monotonically decreasing in *t*.

For proving convergence to stationary points, we note that since *u*(*t*) is of locally bounded variation with no singular part (by assumption), it can be decomposed in an absolutely continuous function and a piecewise constant function [Lebesgue decomposition, see for example Ch. 3 of 47]. As a result, $\dot{u}(t)$ (whenever it exists) is uniformly locally integrable [see 50]. Combined with the fact that *u* is square integrable, which follows from (13) and the observation that *V* is bounded below, we conclude that $u(t)\rightarrow 0$ as $t\rightarrow \infty$ by a variant of Barbalat’s lemma [see 50]. We recall that *x*(*t*) is bounded (*C* is compact) and consider any cluster point $\bar{x}$ of *x*(*t*), which means that there exists a sequence $t_k \rightarrow \infty$ such that $x(t_k) \rightarrow \bar{x}$. We pick any $\tau > 0$ and consider

$$\begin{aligned} u(t_k+\tau )^+-u(t_k)^-&=\int _{t_k}^{t_k+\tau } \textrm{d}u = \int _{t_k}^{t_k+\tau } - 2\delta u - \nabla f(x+\beta u) \textrm{d}t \\  &\quad +\sum _{i=1}^{n_\text {g}} \int _{t_k}^{t_k+\tau } \nabla g_i(x) \textrm{d}\lambda _i, \end{aligned}$$

where we set $\textrm{d}\lambda _i = 0$ whenever $i\not \in I_{x(t)}$ to simplify notation. The previous expression is guaranteed to vanish for $k\rightarrow \infty$, which by continuity of $\nabla g_i$ and $\nabla f$ means that

14 $$\begin{aligned} -\nabla f(\bar{x}) \tau + \sum _{i=1}^{n_\text {g}} \nabla g_i(\bar{x}) \int _{t_k}^{t_k+\tau } \textrm{d}\lambda _i \rightarrow 0 \end{aligned}$$

as $k\rightarrow \infty$. We note that by continuity of *g*, there exists a constant $k_0>0$ and $c_\text {lg}>0$ such that $g_i(x(t_k))>c_\text {lg}$ for all $k>k_0$ and all $i\not \in I_{\bar{x}}$. From the fact that $u(t)\rightarrow 0$ we infer that for large enough *k*, $g_i(x(t))>c_\text {lg}/2$ for all $t\in [t_k,t_k+\tau ]$ and $i\not \in I_{\bar{x}}$. We introduce the notation

$$\begin{aligned} \int _{t_k}^{t_k+\tau } \textrm{d}\lambda _i =: \lambda _k^i \tau , \end{aligned}$$

for all $k>0$ and all $i\in [n_\text {g}]$ and conclude that $\lambda _k^i \ge 0$ and, for all *k* large enough, $\lambda _k^i=0$ if $i\not \in I_{\bar{x}}$.

We argue next that $\lambda _k^i$ is bounded for all $i\in [n_\text {g}]$. We argue by contradiction and consider the sequence $(\lambda _k^1,\dots ,\lambda _k^{n_\text {g}})/|(\lambda _k^1,\dots ,\lambda _k^{n_\text {g}})|$, which is guaranteed to be bounded, even though $|(\lambda _k^1,\dots ,\lambda _k^{n_\text {g}})|\rightarrow \infty$. Upon dividing (14) by $|(\lambda _k^1,\dots ,\lambda _k^{n_\text {g}})|$ we conclude that

$$\begin{aligned} \sum _{i=1}^{n_\text {g}} \nabla g_i(\bar{x}) \xi _i = 0, \end{aligned}$$

where $\xi$ denotes an accumulation point of $(\lambda _k^1,\dots ,\lambda _k^{n_\text {g}})/|(\lambda _k^1,\dots ,\lambda _k^{n_\text {g}})|$, which satisfies $\xi _i \ge 0$, $\xi _i=0$ for all $i\not \in I_{\bar{x}}$, and $|\xi |=1$. However, the fact that $\xi \ne 0$ contradicts the constraint qualification, since it precludes the existence of a vector *w* such that $w^{\mathsf {\tiny {T}}}\nabla g_i(\bar{x})>0$ for all $i\in I_{\bar{x}}$ (which would mean $\sum _{i=1}^{n_\text {g}} w^{\mathsf {\tiny {T}}}\nabla g_i(\bar{x}) \xi _i>0$). This implies that $\lambda _k^i$ is bounded for all $i\in [n_\text {g}]$.

We take any accumulation point of $(\lambda _k^1,\dots ,\lambda _k^{n_\text {g}})$, which we denote by $\bar{\lambda }$. The accumulation point $\bar{\lambda }$ satisfies $\bar{\lambda }\ge 0$, $\bar{\lambda }_i=0$ for all $i\not \in I_{\bar{x}}$ (complementary slackness), and by (14), $-\nabla f(\bar{x}) + \sum _{i=1}^{n_\text {g}} \nabla g_i(\bar{x}) \bar{\lambda }_i = 0$. Hence, $\bar{x}$ and $\bar{\lambda }$ satisfy the Karush-Kuhn-Tucker conditions of (1), and $\bar{x}$ is stationary.

We conclude the proof by showing asymptotic stability of isolated local minima (in the sense of Lyapunov). We accordingly pick any isolated local minimum $x^*$ and note that the function $|u|^2/2 +f(x)-f(x^*)$ is positive definite in a neighborhood of $(x^*,0)$. We conclude from (13) that $x^*$ is therefore stable in the sense of Lyapunov. We have already shown attractiveness (see previous paragraphs) and therefore conclude that $x^*$ is asymptotically stable in the sense of Lyapunov. $\square$

**Table 2:** The table summarizes convergence rates that arise from different choices of $\alpha$, $\beta$, and $\delta$

Variant	$\alpha$	$\delta$	$\beta$	Rate $\rho$
Heavy ball	$\sqrt{\mu }$	$\sqrt{\mu }$	0	$e^{-\sqrt{\mu } t}$
Nesterov constant parameters	$\sqrt{\mu }-\mu /2$	$\frac{\sqrt{\mu }}{1+\sqrt{\mu }}$	$\frac{1-\sqrt{\mu }}{1+\sqrt{\mu }}$	$e^{-(\sqrt{\mu }-\mu /2)t}$
Nesterov varying parameters	$\frac{2}{t+3}$	$\frac{3}{2(t+3)}$	$\frac{t}{t+3}$	$\frac{9}{(t+3)^2}$

Without loss of generality *f* is normalized, such that the Lipschitz constant of the gradient is unity

The following theorem demonstrates that the use of momentum combined with well-chosen damping parameters indeed results in accelerated convergence rates ($\mathcal {O}(1/t^2)$ in the smooth and convex case, and $e^{-\sqrt{\mu } t}$ in the smooth and strongly convex case).^7^

### Theorem 2

Let *C* be convex and *f* be 1-smooth and either convex or strongly convex with strong convexity constant $\mu >0$. Let the parameters $\alpha$, $\beta$, $\delta$, and $\rho$ be chosen according to Table 2 and assume that Slater’s condition holds. Then, for any $x(0)\in \mathbb {R}^{n}$, $u(0)=0$, the following holds:

$$\begin{aligned}  &   \min \{ 0, g(x(0))\} ^{\mathsf {\tiny {T}}}\lambda ^* \rho (t) \\  &   \quad \le f(x(t))-f(x^*) \le \left( \frac{\alpha ^2}{2} |x(0)-x^*|^2 + f(x(0))-f(x^*)\right) \rho (t), \end{aligned}$$

where $x^*$ is a minimizer of (1) and $\lambda ^*$ is a multiplier that satisfies the Karush-Kuhn-Tucker conditions.

### Proof

***(i) Heavy ball*** ($$\alpha =\sqrt{\mu }$$):

We start by analyzing the heavy ball variant ($\alpha =\sqrt{\mu }$) and consider the evolution of the function

$$\begin{aligned} {W}(t)=\frac{1}{2} |\alpha (x(t)-x^*)+u(t)|^2 + f(x(t))-f(x^*), \end{aligned}$$

along the trajectories (*x*(*t*), *u*(*t*)). The corresponding differential measure $\textrm{d}W$ is given by [47]:

$$\begin{aligned} (\alpha (x-x^*)+ (u^+ + u^-)/2 )^{\mathsf {\tiny {T}}}(\alpha u ~\textrm{d}t + \textrm{d}u) + \nabla f(x)^{\mathsf {\tiny {T}}}u ~\textrm{d}t . \end{aligned}$$

By following the same steps as in the proof of Theorem 1 we obtain

$$\begin{aligned} (u^+ + u^-)/2^{\mathsf {\tiny {T}}}\textrm{d}u + \nabla f(x)^{\mathsf {\tiny {T}}}u ~\textrm{d}t \le - 2\delta |u|^2~ \textrm{d}t + \sum _{i\in I_x} -\alpha g_i(x) \textrm{d}\lambda _i. \end{aligned}$$

However, in contrast to Theorem 1, we allow also for infeasible initial conditions, and therefore the term $-\alpha g_i(x) \textrm{d}\lambda _i$ remains. This yields the following upper bound on $\textrm{d}W$:

15 $$\begin{aligned} \begin{array}{c} \textrm{d}W \le -(2\delta -\alpha ) |u|^2 \textrm{d}t - \alpha (x-x^*)^{\mathsf {\tiny {T}}}\nabla f(x) \textrm{d}t +(\alpha ^2-2\alpha \delta ) (x-x^*)^{\mathsf {\tiny {T}}}u \textrm{d}t \\ + {\sum }_{i\in I_x} (\alpha \nabla g_i(x)^{\mathsf {\tiny {T}}}(x-x^*) - \alpha g_i(x)) \textrm{d}\lambda _i. \end{array}\end{aligned}$$

The fact that *f* is strongly convex means that the following holds:

$$\begin{aligned} -(x-x^*)^{\mathsf {\tiny {T}}}\nabla f(x) \le&-(f(x)-f(x^*))-\frac{\mu }{2} |x-x^*|^2. \end{aligned}$$

In addition, each $g_i$ is concave, and therefore

$$\begin{aligned} \nabla g_i(x)^{\mathsf {\tiny {T}}}(x^*-x)\ge g_i(x^*) - g_i(x)\ge -g_i(x), \end{aligned}$$

where we used the fact that $x^*$ is feasible for the last inequality. This concludes that the summand in (15) is non-positive since $\textrm{d}\lambda _i\ge 0$. Thus, after some elementary manipulations, we obtain the following upper bound on $\textrm{d}W$:

$$\begin{aligned} \textrm{d}W \le&-\alpha W \textrm{d}t -(2\delta -3 \alpha /2) |u|^2 \textrm{d}t \le -\alpha W \textrm{d}t . \end{aligned}$$

Applying Grönwall’s inequality then implies the desired upper bound on $f(x(t))-f(x^*)$.

The lower bound is obtained from a perturbation analysis, using an argument similar to Muehlebach and Jordan [1]. We define

$$\begin{aligned} f^*(t):=\min _{\xi \in \mathbb {R}^n} f(\xi ), \quad \text {s.t.} \quad g(\xi )\ge \min \{0,g(x(0))\} e^{-\alpha t}, \end{aligned}$$

which is of the form (1), but with a modified right-hand side of the constraints. The trajectory *x*(*t*) satisfies $g(x(t))\ge \min \{0,g(x(0))\} e^{-\alpha t}$ and is therefore a feasible candidate for the above minimization, which implies $f(x(t))\ge f^*(t)$. The minimum is clearly attained, since *f* is bounded below, $f(x)\rightarrow \infty$ for $|x|\rightarrow \infty$, and the modified set of feasible points is closed. The multiplier $\lambda ^*$ satisfying the Karush-Kuhn-Tucker conditions of (1) captures the sensitivity of the cost function with respect to perturbations of the constraints. This means that $\lambda ^*$ is guaranteed to satisfy the following inequality [see, e.g., 55, RockafellarConvex]:

$$\begin{aligned} f^*(t)-f^* \ge \min \{ 0, g(x(0))\}^{\mathsf {\tiny {T}}}\lambda ^* e^{-\alpha t}, \end{aligned}$$

which combined with $f(x(t))\ge f^*(t)$ implies the desired lower bound.

***(ii) Nesterov-constant parameters*** ($\alpha =\sqrt{\mu }-\mu /2$):

We consider again the evolution of the function *W*(*t*) (see (i)) along the trajectories (*x*(*t*), *u*(*t*)). The corresponding differential measure is given by

$$\begin{aligned} \textrm{d}W= ({\alpha } (x-x^*)+ (u^+ + u^-)/2 )^{\mathsf {\tiny {T}}}({\alpha } u \textrm{d}t + \textrm{d}u) + \nabla f(x)^{\mathsf {\tiny {T}}}u \textrm{d}t . \end{aligned}$$

From the proof of Theorem 1 we obtain16Including the remaining terms yields the following upper bound on $\textrm{d}W$:

17 $$\begin{aligned}&-(2\delta -{\alpha }) |u|^2 \textrm{d}t + ({\alpha }^2 - 2\delta a) (x-x^*)^{\mathsf {\tiny {T}}}u \textrm{d}t \nonumber \\&\qquad - (\nabla f(y) - \nabla f(x))^{\mathsf {\tiny {T}}}u \textrm{d}t - {\alpha } (x-x^*)^{\mathsf {\tiny {T}}}\nabla f(y) \textrm{d}t \nonumber \\&\qquad + \sum _{i\in I_x} (a \nabla g_i(x)^{\mathsf {\tiny {T}}}(x-x^*) - \alpha g_i) \textrm{d}\lambda _i, \end{aligned}$$

where we introduced the variable $y:=x+\beta u$ to simplify notation. The fact that *f* is strongly convex means that the following holds:

18 $$\begin{aligned} -(x-x^*)^{\mathsf {\tiny {T}}}\nabla f(y)&\le -(f(x)-f(x^*)) -\beta (\nabla f(x)-\nabla f(y))^{\mathsf {\tiny {T}}}u - \mu \beta ^2 |u|^2 \nonumber \\&\quad - \mu \beta u^{\mathsf {\tiny {T}}}(x-x^*) -\frac{\mu }{2} |x-x^*|^2. \end{aligned}$$

In addition, *C* is convex, which implies that each $g_i$ is concave. As a result,

$$\begin{aligned} \nabla g_i(x)^{\mathsf {\tiny {T}}}(x^*-x)\ge g_i(x^*) - g_i(x)\ge -g_i(x), \end{aligned}$$

where we used the fact that $x^*$ is feasible for the last inequality. The summand in (17) can therefore be upper bounded by

$$\begin{aligned} \left( {\alpha } \nabla g_i(x)^{\mathsf {\tiny {T}}}(x-x^*) - \alpha g_i(x)\right) \textrm{d}\lambda _i \le 0, \end{aligned}$$

since, by definition $\textrm{d}\lambda _i\ge 0$. Combined with (18), and after some elementary manipulations, this yields the following upper bound on $\textrm{d}W$:

$$\begin{aligned} \textrm{d}W&\le -{\alpha } W \textrm{d}t -(2\delta -3{\alpha }/2 +\mu \beta ) |u|^2 \textrm{d}t - \frac{1}{2} ({\alpha } \mu - {\alpha }^3) |x-x^*|^2 \textrm{d}t \\&\quad + (2 {\alpha }^2 - 2 \delta {\alpha } - {\alpha } \mu \beta ) (x-x^*)^{\mathsf {\tiny {T}}}u \textrm{d}t , \end{aligned}$$

where we have used the fact that $1-\beta {\alpha }\ge 0$. We note that the term $2{\alpha }^2-2\delta {\alpha }-{\alpha }\mu \beta$ vanishes, that ${\alpha }(\mu -{\alpha }^2)\ge 0$, and $2\delta -3{\alpha }/2+\mu \beta \ge 0$ for all $\mu \in [0,1]$. We therefore obtain $\textrm{d}W\le -{\alpha } W \textrm{d}t$, which, by Grönwall’s inequality, implies the desired result.

***(iii) Nesterov - varying parameters*** (${\alpha (t)}=2/(t+3)$):

The proof follows the same steps. We consider again the evolution of the function

$$\begin{aligned} {W(t)}=\frac{1}{2} |{\alpha (t)}(x(t)-x^*) + u(t)|^2+f(x(t))-f(x^*), \end{aligned}$$

along the trajectories (*x*(*t*), *u*(*t*)), where $\alpha (t)$ is now time-varying, and where we will again omit the dependence with respect to *t* to simplify notation. The differential measure $\textrm{d}{W}$ is given by

$$\begin{aligned} \textrm{d}{W}= ({\alpha } (x-x^*)+ (u^+ + u^-)/2 )^{\mathsf {\tiny {T}}}({\alpha } u \textrm{d}t + {\dot{\alpha }} (x-x^*) \textrm{d}t + \textrm{d}u) + \nabla f(x)^{\mathsf {\tiny {T}}}u \textrm{d}t , \end{aligned}$$

where according to the chain rule the derivative of ${\alpha }$ with respect to *t* enters. This leads to two additional quadratic terms of the type

$$\begin{aligned} {\alpha } {\dot{\alpha }} |x-x^*|^2 \textrm{d}t \quad \text {and}\quad {\dot{\alpha }} u^{\mathsf {\tiny {T}}}(x-x^*)\textrm{d}t . \end{aligned}$$

As a result, by following the same steps as in the variant (ii) (see previous section), we obtain

$$\begin{aligned} \textrm{d}{W}&\le -{\alpha } {W} \textrm{d}t -(2\delta -3{\alpha }/2 +\mu \beta ) |u|^2 \textrm{d}t - \frac{1}{2} ({\alpha } \mu - {\alpha }^3 - 2{\alpha } {\dot{\alpha }}) |x-x^*|^2 \textrm{d}t \\&\quad + (2 {\alpha }^2 - 2 \delta {\alpha } - {\alpha } \mu \beta +{\dot{\alpha }}) (x-x^*)^{\mathsf {\tiny {T}}}u \textrm{d}t . \end{aligned}$$

We note that $2{\alpha }^2-2\delta {\alpha } + {\dot{\alpha }}$ vanishes. The same applies to $2\delta -3{\alpha }/2$ and ${\alpha }^3-2{\alpha }{\dot{\alpha }}$, which simplifies the above inequality to

$$\begin{aligned} \textrm{d}{W}\le -{\alpha } {W} \textrm{d}t -\mu \beta |u|^2 \textrm{d}t - \frac{1}{2} {\alpha } \mu |x-x^*|^2 \textrm{d}t - {\alpha } \mu \beta (x-x^*)^{\mathsf {\tiny {T}}}u \textrm{d}t . \end{aligned}$$

Applying Young’s inequality to the cross-term $(x-x^*)^{\mathsf {\tiny {T}}}u$ concludes that $\textrm{d}{W} \le -{\alpha } {W} \textrm{d}t$ for any $\mu \in [0,1]$. We finally apply Grönwall’s inequality, which yields

$$\begin{aligned} {W(t)} \le {W(0)} \exp \left( -\int _{0}^{t} {\alpha }(s) \textrm{d}s\right) ={W(0)} \frac{9}{(t+3)^2}, \end{aligned}$$

and concludes the proof. $\square$

We also demonstrate convergence of the discrete algorithm (8) in a nonconvex and possibly stochastic setting. For simplicity we state and prove the deterministic result when $\epsilon =0$ (as becomes apparent from the proof, the stochastic case with bounded zero-mean gradient perturbations follows from the same arguments). We further note that the restriction $1/2<s<1$ can be loosened to $1/2<s\le 1$ if additional assumptions on the damping parameters $\delta$ and $\beta$ are satisfied (this requires a slightly more detailed proof).

### Theorem 3

Let $T_k=T_0/k^s$, $k=1,2,\dots$, for some $T_0>0$ and $s\in (1/2,1)$, and let the function $\min \{0,g_1(x)\}$ have compact level sets. Let *f* be 1-smooth and either convex or such that $2\delta -\beta >0$, let $x_k,u_k$ be the iterates defined in (8) with $\epsilon =0$ and the initial values $(x_0,u_0)\in \mathbb {R}^{2n}$ arbitrary, and let *g* satisfy the Mangasarian-Fromovitz constraint qualification. If $u_k$ is bounded and *f* has isolated stationary points, then $x_k$ converges to a stationary point of (1), while $u_k$ converges to zero.

### Proof

The update (8) can be divided into the following two steps

19 $$\begin{aligned} \bar{x}_k&= x_k, \qquad \qquad \qquad \qquad \qquad ~~~~ x_{k+1}=\bar{x}_k + u_{k+1} T_k, \nonumber \\ \bar{u}_k&= u_k - T_k f_\text {d}(x_k,u_k) + R_k, \qquad u_{k+1}=\bar{u}_k - T_k \nabla f(\bar{x}_k), \end{aligned}$$

where $f_\text {d}(x_k,u_k):=2 \delta u_k + \nabla f(x_k + \beta u_k) - \nabla f(x_k)$ contains the dissipative terms and $R_k$ denotes the constraint forces. The first step, which maps $(x_k,u_k)$ to $(\bar{x}_k, \bar{u}_k)$ is an update of the velocity with the dissipative terms and the constraint forces $R_k$, whereas the second step, which maps $(\bar{x}_k, \bar{u}_k)$ to $(x_{k+1},u_{k+1})$ is a symplectic Euler discretization that captures the conservative parts of the underlying dynamics. In the following we will exploit the fact that the second step is a symplectic map. More precisely, the fact that the second step is symplectic implies that an energy function $V_k: \mathbb {R}^n\times \mathbb {R}^n \rightarrow \mathbb {R}$ (bounded below, uniformly in *k*) exists, such that

$$\begin{aligned} V_{k+1}(x_{k+1},u_{k+1}) - V_k(x_k,u_k) \le - c_{\text {V}1} T_k |u_k|^2 - c_{\text {V}2} |R_k|^2 + c_{\text {V}3} T_k^2 - \alpha \sum _{i\in I_{x_k}} \lambda _k^i g_i (x_k) \end{aligned}$$

for $k\ge k_0$ and for some constants $k_0>0$, $c_{\text {V}1}, c_{\text {V}2}, c_{\text {V}3} > 0$. A detailed derivation of this step is included in Appendix D (see Lemma 12). Furthermore, as a result of the diminishing step-size, we infer that $g_i(x_k)\ge -c_\text {g} T_k$ holds for all sufficiently large *k* and a constant $c_\text {g}>0$ (see Lemma 9 in Appendix D). We can therefore bound the terms $\lambda _k^i g_i(x_k)$ as follows

20 $$\begin{aligned} |-\alpha \sum _{i\in I_{x_k}} \lambda _k^i g_i (x_k)| \le \frac{\alpha }{c_\lambda } c_\text {g} T_k |R_k| n_\text {g} \le \frac{c_{\text {V}2}}{2} |R_k|^2 + \frac{1}{ 2 c_{\text {V}2}} (n_\text {g} c_\text {g} \frac{\alpha }{c_\lambda })^2 T_k^2, \end{aligned}$$

where we have exploited constraint qualification to conclude that $|\lambda _k|\le |R_k|/c_\lambda$ (see Lemma 10 in Appendix D) in the first step, and we have used Young’s inequality in the second step. We therefore obtain

21 $$\begin{aligned} V_{k+1}(x_{k+1},u_{k+1}) - V_k(x_k,u_k) \le - c_{\text {V}1} T_k |u_k|^2 - \frac{c_{\text {V}2}}{2} |R_k|^2 + \bar{c}_{\text {V}3} T_k^2, \end{aligned}$$

for large enough *k* and a modified constant $\bar{c}_{\text {V}3}>0$. We further introduce the constants

$$\begin{aligned} -c_{\text {V}0} = \inf _k V_k(x_k,u_k), \qquad c_{\text {T}1}= \sum _{k=k_0}^{\infty } T_k^2, \end{aligned}$$

and make the following claim: There exists a subsequence $k(j), j=1,2,\dots$, such that

$$\begin{aligned} -c_{\text {V}1} T_{k(j)} |u_{k(j)}|^2 - \frac{c_{\text {V}2}}{2} |R_{k(j)}|^2 \ge \underbrace{\left( -\bar{c}_{\text {V}3} - \frac{V_{k_0}(x_{k_0},u_{k_0})+c_{\text {V}0}}{c_{\text {T}1}} - \frac{1}{c_{\text {T}1}} \right) }_{:=-c_{\text {V}4}} T_{k(j)}^2, \end{aligned}$$

where $c_{\text {V}4}>0$. For the sake of contradiction we assume that the claim is not true, which means that

$$\begin{aligned} -c_{\text {V}1} T_{k} |u_{k}|^2 - \frac{c_{\text {V}2}}{2} |R_{k}|^2 \le - {c}_{\text {V}4} T_{k}^2 \end{aligned}$$

for all $k>0$. However, we have chosen the constant $c_{\text {V}4}$ deliberately in such a way that we can generate a contradiction when summing over $V_{k+1}(x_{k+1},u_{k+1})-V_{k}(x_k,u_k)$. More precisely,

$$\begin{aligned} -c_{\text {V}0} - V_{k_0}(x_{k_0},u_{k_0})&\le V_N(x_N,u_N) - V_{k_0}(x_{k_0},u_{k_0}) \le \sum _{k=k_0}^{N} (-c_{\text {V}4} + \bar{c}_{\text {V}3}) T_k^2 \\&\le \frac{-c_{\text {V}0} - V_{k_0}(x_{k_0},u_{k_0}) -1 }{c_{\text {T}1}} \sum _{k=k_0}^{N} T_k^2, \end{aligned}$$

which leads to the desired contradiction since the right-hand side approaches $-c_{\text {V}_0} - V_{k_0}(x_{k_0},u_{k_0}) - 1$ for $N\rightarrow \infty$.

Upon passing to another subsequence we infer that $u_{k(j)} \rightarrow 0$, $R_{k(j)}/T_{k(j)} \rightarrow \bar{R}$, and $x_{k(j)} \rightarrow \bar{x}\in C$. By the properties of the set $V_\alpha (x)$ we conclude that $\bar{x}$ is stationary and $\bar{x}$ and $\bar{R}$ satisfy the Karush-Kuhn-Tucker conditions, see Lemma 11 in Appendix D.

We now prove that the entire sequence converges. We infer from (21) and [52, Lemma 2, Sec. 2.2.1 ] that the sequence $V_k(x_k,u_k)$ converges and satisfies

$$\begin{aligned} \lim _{k\rightarrow \infty } V_k(x_k,u_k)= \lim _{j\rightarrow \infty } \left( \frac{1}{2}|u_{k(j)}|^2 + f(x_{k(j)}) \right) = f(\bar{x}), \end{aligned}$$

where $\bar{x}$ is the limit of $x_{k(j)}$ (see above). We further introduce the collection of all accumulation points of $x_k,u_k$, that is,

$$\begin{aligned} \omega := \bigcap _{m\ge 0} \text {cl}\{(x_k,u_k):k>m\}, \end{aligned}$$

where $\text {cl}$ denotes closure. We note that $\omega \subset C \times \mathbb {R}^n$ is connected (since $|x_{k+1}-x_k| \rightarrow 0$ and $|u_{k+1}-u_k|\rightarrow 0$) and for any $(x,u)\in \omega$, $|u|^2/2+f(x)=f(\bar{x})$.

We claim that $\omega =\{(\bar{x},0)\}$. For the sake of contradiction, we assume the existence of a sequence $(\tilde{x}_k, \tilde{u}_k) \rightarrow {(\tilde{x},\tilde{u})}$, $(\tilde{x}_k,\tilde{u}_k)\in \omega$ with $(\tilde{x},\tilde{u}) \ne (\bar{x},0)$ ($\omega$ is connected). We consider first the case where $\bar{x}$ lies in the interior of *C* (constraints are not active). In that case $\tilde{x}_k$ lies likewise in the interior of *C* for large *k*. However, in the absence of constraints the dynamics in (8) are smooth, which means that $\tilde{x}_k$ and $\tilde{u}_k$ are guaranteed to be equilibria and therefore satisfy $\tilde{u}_k=0$ and $\nabla f(\tilde{x}_k)=0$. This contradicts the fact that *f* has isolated stationary points. Next, we consider the case where $\bar{x}$ lies on the boundary of *C*, where we infer from $(\tilde{x},\tilde{u})\in \omega$ that $f(\tilde{x})=f(\bar{x})-|\tilde{u}|^2/2$. Therefore $f(\tilde{x}) \le f(\bar{x})$, $\tilde{x}\in C$, which also contradicts the fact that $\bar{x}$ is an isolated stationary point.

Thus, we conclude $\omega =\{(\bar{x},0)\}$ and the result follows. $\square$

We note that the behavior of algorithm (8) is complex, as it relies on a *local* approximation of the feasible set, whereby multiple constraints can become active or inactive over the course of the optimization. Establishing Theorem 3 is therefore nontrivial and requires blending ideas from numerical analysis, optimization, and dynamical systems.

We conclude the section by proving an accelerated rate for a slightly modified version of (8) in a convex setting. The algorithm we will consider is the following: 
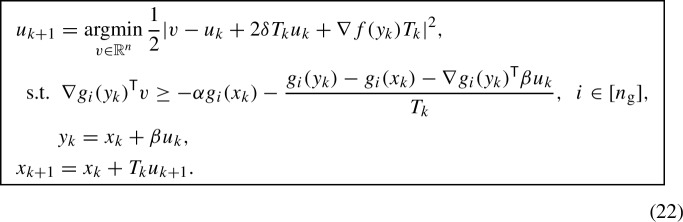


There are a few differences between (8) and (22). Instead of evaluating $\nabla g$ at $x_k$, the gradient is evaluated at $x_k+\beta u_k$, which will simplify our proof (both $\nabla f$ and $\nabla g$ are evaluated at the same point). Furthermore the right-hand side of the inequality constraint includes an additional term that can be related to the curvature of $g_i$ as follows

$$\begin{aligned} -\frac{g_i(y_k)-g_i(x_k)-\nabla g_i(y_k)^{\mathsf {\tiny {T}}}\beta u_k}{T_k} = \frac{\nabla ^2 g_i({\xi _k^i}) \beta ^2 |u_k|^2}{2T_k}, \end{aligned}$$

for some ${\xi _k^i}$ between $x_k$ and $y_k$, where Taylor’s theorem has been used in the second step. These modifications simplify the analysis and will enable us to derive accelerated non-asymptotic convergence rates for the discrete algorithm (22). We conjecture (based on numerical experiments, see Sect. 5) that the modification of the right-hand side of the inequality constraints in (22) is an artifact of our analysis.

### Theorem 4

Let *f* be $\mu$-strongly convex and let *g* be smooth and concave. Let $l(x)=f(x)-{\lambda ^*}^{\mathsf {\tiny {T}}}g(x)$ denote the Lagrangian, where $\lambda ^*$ denotes an optimal multiplier of (1). Then, the iterates of (22) with $T_k=1/\sqrt{L_l}$, $\delta =\sqrt{L_l}/(\sqrt{\kappa _l}+1)$, $\beta =T_k(1-2\delta T_k)$, $\alpha =\delta$, and $u_0=0$ satisfy:

$$\begin{aligned} \frac{\mu }{2} |x_k-x^*|^2 \le l(x_k)-l(x^*)\le \left( 1-\frac{1}{1+\sqrt{\kappa _l}}\right) ^k \left( \frac{L_l}{8} |x_0-x^*|^2 + l(x_0)-l(x^*)\right) , \end{aligned}$$

for all $k\ge 0$, where $L_l$ denotes the smoothness constant of *l* and $\kappa _l=L_l/\mu$.

### Proof

We simplify the presentation by assuming that $L_l={T_k}=1$, which is without loss of generality. The proof hinges on the following Lyapunov function:

$$\begin{aligned} V(x,u):=\frac{1}{2} |\delta (x-x^*) + (1-\delta ) u|^2+ l(x)-l(x^*), \end{aligned}$$

which can be readily verified to be positive definite for all $\kappa _l\ge 1$. Moreover, the evolution of *V* along the iterates (22) is given by

$$\begin{aligned} V_{k+1}-V_k= &   \frac{1}{2} |\delta (x_{k+1}-x_k)+ (1-\delta ) (u_{k+1}-u_k)|^2 + l(x_{k+1})-l(x_k)\\  &   + (\delta (x_{k+1}-x_k)+ (1-\delta ) (u_{k+1}-u_k))^{\mathsf {\tiny {T}}}(\delta (x_k-x^*)+(1-\delta ) u_k), \end{aligned}$$

where we abbreviated $V(x_k,u_k)$ by $V_k$. We further rewrite $u_{k+1}=(1-2\delta ) u_k-\nabla \bar{l}(y_k)$, with $\nabla \bar{l}(y_k):=\nabla f(y_k)- \nabla g(y_k)\lambda _k$ and $\lambda _k$ an optimal multiplier of (22) (see also (24) below). This implies that $\delta (x_{k+1}-x_k)+ (1-\delta ) (u_{k+1}-u_k)=u_{k+1}-(1-\delta )u_k=-\delta u_k - \nabla \bar{l}(y_k)$ and therefore

$$\begin{aligned} V_{k+1}-V_k= &   \frac{1}{2} |\delta u_k + \nabla \bar{l}(y_k)|^2 + l(x_{k+1})-l(x_k)\\  &   +(-\delta u_k - \nabla \bar{l}(y_k))^{\mathsf {\tiny {T}}}(\delta (x_k-x^*) + (1-\delta ) u_k). \end{aligned}$$

By expanding the terms and exploiting that $\beta =1-2\delta$ we arrive at

23 $$\begin{aligned} V_{k+1}-V_k&= -\delta (1-\frac{3\delta }{2}) |u_k|^2 -\beta u_k^{\mathsf {\tiny {T}}}\nabla \bar{l}(y_k)+\delta (x^*-x_k)^{\mathsf {\tiny {T}}}\nabla \bar{l}(y_k)\nonumber \\&\quad +\delta ^2 u_k^{\mathsf {\tiny {T}}}(x^*-x_k) +\frac{1}{2} |\nabla \bar{l}(y_k)|^2+l(x_{k+1})-l(x_k). \end{aligned}$$

Next, we will relate $l(x_{k+1})$ to $l(y_k)$. To do so, we start by slightly reformulating the minimization in (22) by performing a change of variables, $x=x_k+T_k v$, which yields

24 $$\begin{aligned} x_{k+1}&={\mathop {\textrm{argmin}}\limits _{x\in \mathbb {R}^n}} \frac{1}{2} |x-y_k+\nabla f(y_k)|^2\nonumber \\&\text {s.t.}~\nabla g_i(y_k)^{\mathsf {\tiny {T}}}(x-y_k) \ge (1-\alpha T_k) (g_i(x_k)-g_i(y_k))-\alpha T_k g_i(y_k), ~~i\in [n_\text {g}], \nonumber \\ \end{aligned}$$

where we have used the fact that $T_k(1-2\delta T_k)=\beta$ to simplify the objective function. We note that $x=\alpha (x^*-x_k)+x_k$ is a feasible solution candidate in the minimization (24). This follows from the following reasoning:

$$\begin{aligned} \nabla g_i(y_k) (\alpha (x^*-x_k)+x_k-y_k)&=(1-\alpha )\nabla g_i(y_k)^{\mathsf {\tiny {T}}}(x_k-y_k)+\alpha \nabla g_i(y_k)^{\mathsf {\tiny {T}}}(x^*-y_k)\\&\ge (1-\alpha ) (g_i(x_k)-g_i(y_k)) + \alpha (g_i(x^*)-{g_i}(y_k)), \end{aligned}$$

where concavity of *g* is used in the second step together with the fact that $T_k=1$ and $g_i(x^*)\ge 0$. As a result, the stationarity condition of (24) implies

$$\begin{aligned} (x_{k+1}-y_k+\nabla f(y_k))^{\mathsf {\tiny {T}}}(\alpha (x^*-x_k)+x_k-x_{k+1})\ge 0. \end{aligned}$$

This inequality can be restated as follows:

$$\begin{aligned}&(x_{k+1}-y_k+\nabla f(y_k))^{\mathsf {\tiny {T}}}(\alpha (x^*-x_k)-\beta u_k -(x_{k+1}-y_k))\\&\quad = - |\nabla \bar{l}(y_k)|^2 -\nabla \bar{l}(y_k)^{\mathsf {\tiny {T}}}(\alpha (x^*-x_k)-\beta u_k) \\&\qquad + \nabla f(y_k)^{\mathsf {\tiny {T}}}(\alpha (x^*-x_k) -\beta u_k) - \nabla f(y_k)^{\mathsf {\tiny {T}}}(x_{k+1}-y_k) \ge 0. \end{aligned}$$

Rearranging this inequality yields the following bound:

$$\begin{aligned} \nabla l(y_k)^{\mathsf {\tiny {T}}}(x_{k+1}-y_k)\le &   - |\nabla \bar{l}(y_k)|^2 -\nabla \bar{l}(y_k)^{\mathsf {\tiny {T}}}(\alpha (x^*-x_k)-\beta u_k) \nonumber \\  &   + \nabla f(y_k)^{\mathsf {\tiny {T}}}(\alpha (x^*-x_k) -\beta u_k) -{\lambda ^*}^{\mathsf {\tiny {T}}}\nabla g(y_k)^{\mathsf {\tiny {T}}}(x_{k+1}-y_k). \end{aligned}$$

As a result of this inequality and the smoothness of *l*, we can bound the evolution of $l(x_{k+1})$ as

25 $$\begin{aligned} l(x_{k+1})&\le l(y_k)+ \nabla l(y_k)^{\mathsf {\tiny {T}}}(x_{k+1}-y_k) +\frac{1}{2} |\nabla \bar{l}(y_k)|^2\nonumber \\&\le l(y_k) - \frac{1}{2} |\nabla \bar{l}(y_k)|^2-\nabla \bar{l}(y_k)^{\mathsf {\tiny {T}}}(\alpha (x^*-x_k)-\beta u_k) \nonumber \\&\quad + \nabla f(y_k)^{\mathsf {\tiny {T}}}(\alpha (x^*-x_k) -\beta u_k) -{\lambda ^*}^{\mathsf {\tiny {T}}}\nabla g(y_k)^{\mathsf {\tiny {T}}}(x_{k+1}-y_k). \end{aligned}$$

By combining (25) with (23) we obtain

26 $$\begin{aligned} V_{k+1}-V_k&\le -\delta (1-\frac{3\delta }{2}) |u_k|^2 +\nabla f(y_k)^{\mathsf {\tiny {T}}}(\alpha (x^*-x_k) -\beta u_k) +l(y_k)-l(x_k) \nonumber \\&\quad -{\lambda ^*}^{\mathsf {\tiny {T}}}\nabla g(y_k)^{\mathsf {\tiny {T}}}(x_{k+1}-y_k) +\delta ^2 u_k^{\mathsf {\tiny {T}}}(x^*-x_k), \end{aligned}$$

where we have exploited that $\alpha =\delta$ and the fact that all the terms containing $\nabla \bar{l}(y_k)$ cancel out (in fact the Lyapunov function and the algorithm (22) are specifically engineered in this way). The strong convexity of *f* implies that

27 $$\begin{aligned} \nabla f(y_k)^{\mathsf {\tiny {T}}}(\delta (x^*-x_k) - \beta u_k)\le &   -\delta (f(x_k)-f(x^*)) - \frac{\delta \mu }{2} |x^*-x_k|^2 - \delta \beta \mu (x_k-x^*)^{\mathsf {\tiny {T}}}u_k \nonumber \\  &   - \frac{\mu }{2} \beta ^2 |u_k|^2 +f(x_k)-f(y_k); \end{aligned}$$

see Muehlebach and Jordan [8, App. A6], which yields, combined with (26),

$$\begin{aligned}&V_{k+1}-V_k\le -\delta (1-\frac{3\delta }{2}+\frac{\beta ^2\mu }{2\delta }) |u_k|^2- \frac{\delta \mu }{2} |x^*-x_k|^2 + \delta (\beta \mu +\delta ) (x^*-x_k)^{\mathsf {\tiny {T}}}u_k \\&\quad -\delta (f(x_k)-f(x^*)) -{{\lambda ^*}^{\mathsf {\tiny {T}}}} g(y_k) + {\lambda ^*}^{\mathsf {\tiny {T}}}g(x_k) -{\lambda ^*}^{\mathsf {\tiny {T}}}\nabla g(y_k)^{\mathsf {\tiny {T}}}(x_{k+1}-y_k). \end{aligned}$$

This can be rearranged as follows:

$$\begin{aligned} V_{k+1}-V_k\le &   -\delta V_k -\frac{1}{2} (\delta -\delta ^2-\delta ^3+\beta ^2\mu ) |u_k|^2\\  &   - \frac{\delta \mu -\delta ^3}{2} |x^*-x_k|^2 + \delta (\beta \mu +\delta ^2) (x^*-x_k)^{\mathsf {\tiny {T}}}u_k\\  &   +(1-\delta ) {\lambda ^*}^{\mathsf {\tiny {T}}}g(x_k)-{\lambda ^*}^{\mathsf {\tiny {T}}}g(y_k) -{\lambda ^*}^{\mathsf {\tiny {T}}}\nabla g(y_k)^{\mathsf {\tiny {T}}}(x_{k+1}-y_k). \end{aligned}$$

We further note that the inequality constraint in (24) implies

$$\begin{aligned} -\nabla g_i(y_k)^{\mathsf {\tiny {T}}}(x_{k+1}-y_k)+(1-\delta ) g(x_k)-g(y_k)\le 0, \end{aligned}$$

and therefore we obtain

$$\begin{aligned} V_{k+1}-V_k&\le -\delta V_k -\frac{1}{2} (\delta -\delta ^2-\delta ^3+\beta ^2\mu ) |u_k|^2\\  &\quad - \frac{\delta \mu -\delta ^3}{2} |x^*-x_k|^2 + \delta (\beta \mu +\delta ^2) (x^*-x_k)^{\mathsf {\tiny {T}}}u_k. \end{aligned}$$

We note that $\beta \mu =(1-\mu ) \delta ^2$ and therefore $\beta \mu +\delta ^2=\delta ^2 (2-\mu )$. We now apply Young’s inequality to the term $(x^*-x_k)^{\mathsf {\tiny {T}}}u_k$ and conclude

$$\begin{aligned} -\frac{1}{2}(\delta -\delta ^2-\delta ^3+\beta ^2\mu ) |u_k|^2- \frac{\delta \mu -\delta ^3}{2} |x^*-x_k|^2 + \delta ^3 (2-\mu ) (x^*-x_k)^{\mathsf {\tiny {T}}}u_k\le 0, \end{aligned}$$

for all $\kappa _l\ge 1$, $x^*-x_k$, and $u_k$. This yields $V_{k+1}-V_k\le -\delta V_k$ and concludes the proof. $\square$

Theorem 4 requires knowledge of the smoothness constant of *l*, which can be restrictive for certain applications. However, by following the same argument as in Appendix A we obtain the following corollary that proves a $\mathcal {O}(\log (1/\varepsilon )/\sqrt{\varepsilon })$ convergence rate if $L_l$ is unknown.

### [Style2 Style1]Corollary 5

Let the function *f* be $\mu$-strongly convex and *L*-smooth, let *g* be concave and $L_\text {g}$-smooth, and let

$$\begin{aligned} B:=\max _{x\in C} |\nabla f(x)|^2/(2\mu ). \end{aligned}$$

Then, the iterate $x_N$ of (22) with $T_k=1/\sqrt{L_l}$, $\delta =\sqrt{L_l}/(\sqrt{\kappa _l}+1)$, $\beta =T_k(1-2\delta T_k)$, $\alpha =\delta$, and $L_l=L+B L_\text {g}/\varepsilon$ satisfies $|x_N-x^*|\le \epsilon$, where

$$\begin{aligned} N&\ge 2\sqrt{\frac{L\!+\!B L_\text {g}/\varepsilon }{\mu }} \!\left( \!\!2\log (1/\varepsilon ) \!+\! \log \left( \frac{5L\!+\!5B L_\text {g}/\varepsilon }{4\mu }\right) \!+\! 2 \log (|x^*-x_0|)\!\right) \\  &= \mathcal {O}\!\left( \!\frac{\log (1/\varepsilon )}{\sqrt{\varepsilon }}\!\right) , \end{aligned}$$

and $x^*$ denotes the minimizer of $\min _{x\in \mathbb {R}^n} f(x)~\text {s.t.}~g(x)\ge - \varepsilon$.

Theorem 4 further requires strong convexity of the objective function. We also obtain an accelerated rate when *f* is convex and smooth, but not strongly convex. This is summarized in the following corollary, which adds a small, but well-chosen, regularization to *f* to render the problem strongly convex.

### [Style2 Style1]Corollary 6

Let the objective function *f* be convex and *L*-smooth, let *g* be concave and $L_g$-smooth, and let $\nabla ^2 f(x^*)-\sum _{i=1}^{n_g}\lambda ^*_i \nabla ^2 g_i(x^*)$ be positive definite, where $\lambda ^*$ denotes an optimal multiplier of (1). Let $L_l$ denote the smoothness constant of the Lagrangian $l(x)=f(x)-g(x)^{\mathsf {\tiny {T}}}\lambda ^*$ and consider the modified objective function

$$\begin{aligned} f_\mu (x)=f(x)+\mu |x-x_0|^2/2\quad \text {with} \quad \mu =\frac{16 L_l \textrm{log}(N)^2}{N^2}. \end{aligned}$$

Then, the iterate $x_N$ of (22), when applied to $f_\mu$ with $\kappa _l=L_l/\mu$, $T_k=1/\sqrt{2L_l}$, $\delta =\sqrt{2L_l}/(\sqrt{2\kappa _l}+1)$, $\beta =T_k(1-2\delta T_k)$, $\alpha =\delta$, and $u_0=0$ satisfies

$$\begin{aligned} l(x_N)-l(x^*) \le \mathcal {O}\left( \frac{\textrm{log}(N)^2}{N^2}\right) . \end{aligned}$$

### Proof

We consider the regularized Lagrangian $l_\mu (x):=f_\mu (x)-g(x)^{\mathsf {\tiny {T}}}\lambda _\mu ^*$, where $\lambda _\mu ^*$ denotes the optimal multiplier of (1) with *f* replaced by $f_\mu$, and define $\lambda ^*:=\lim _{\mu \downarrow 0} \lambda _\mu ^*$. We further introduce the function $d(\lambda ,\mu ):=\min _{x\in \mathbb {R}^n} f_\mu (x)-\lambda ^{\mathsf {\tiny {T}}}g(x)$ and note that $\nabla _\lambda d(\lambda ,\mu )$ is given by

$$\begin{aligned} \nabla _\lambda d(\lambda ,\mu )=-g(x_\mu ^*), \quad \text {where }x_\mu ^*\text { is defined via}~ \nabla f_\mu (x_\mu ^*)-\nabla g(x_\mu ^*) \lambda =0. \end{aligned}$$

Due to the fact that $H:=\nabla ^2 f(x^*)-\sum _{i=1}^{n_g} \lambda _i^* \nabla ^2 g_i(x^*)$ is positive definite, we conclude from the implicit function theorem that $x^*_\mu$ is a continuously differentiable function of $\mu$ and $\lambda$ in a neighborhood of $\mu =0$, $\lambda =\lambda ^*$, and has derivatives

$$\begin{aligned} \frac{\partial x^*_{\mu }}{\partial \lambda }=H^{-1} \nabla g(x^*), \qquad \frac{\partial x^*_{\mu }}{\partial \mu } = -H^{-1} (x^*-x_0) \end{aligned}$$

at $\mu =0, \lambda =\lambda ^*$. This means that $\nabla _\lambda d(\lambda ,\mu )$ is continuously differentiable in both arguments and that $\nabla ^2_\lambda d(\lambda ,\mu )$ is strictly negative definite in a neighborhood around $\mu =0$, $\lambda =\lambda ^*$. We now apply Robinson’s implicit function theorem, see, e.g., [53, Thm. 2B.1], which yields

$$\begin{aligned} |\lambda _\mu ^*-\lambda ^*| \le |x^*-x_0|c_1 \mu , \end{aligned}$$

for all $\mu$ in a neighborhood of the origin and where $c_1\ge 0$ is constant (the scaling with $|x^*-x_0|$ arises from the derivative $\partial x_\mu ^*/\partial \mu$). This means that for large enough *N*, the modified Lagrangian $l_\mu$ is $2L_l$-smooth. Hence, as a result of Theorem 4, we conclude

$$\begin{aligned} l_\mu (x_N)-l_\mu (\tilde{x}^*) \le \exp \left( -\frac{1}{1+\sqrt{2\kappa _l}} N\right) \left( \frac{5L_l}{4} |x_0-\tilde{x}^*|^2\right) , \end{aligned}$$

where we have used the fact that $e^{-\xi } \ge 1-\xi$ for all $\xi \in \mathbb {R}$, as well as the $2L_l$-smoothness of $l_\mu$. In addition, $\tilde{x}^*$ denotes the minimizer of (1) with *f* replaced by $f_\mu$. As a result of the choice of $\mu$ we note that $\sqrt{2\kappa _l}=N/(2\textrm{log}(N)\sqrt{2})$ and $1+\sqrt{2\kappa _l}\le N/(2\textrm{log}(N))$ for large *N*. Hence, we can simplify the right-hand side of the previous inequality for large *N* as follows

$$\begin{aligned} l_\mu (x_N)-l_\mu (\tilde{x}^*) \le \exp \left( -2\textrm{log}(N) \right) \left( \frac{5L_l}{4} |x_0-\tilde{x}^*|^2\right) \le \frac{1}{N^2} \left( \frac{5L_l}{4} |x_0-\tilde{x}^*|^2\right) . \end{aligned}$$

We now relate $l_\mu$ to *l* in the following way:

$$\begin{aligned} (f(x_N)-g(x_N)^{\mathsf {\tiny {T}}}\lambda _\mu ^*)-(f(x^*)-g(x^*)^{\mathsf {\tiny {T}}}\lambda _\mu ^*)&\le l_\mu (x_N) - l_\mu (x^*) + \frac{\mu }{2} |x^*-x_0|^2\\&\le l_\mu (x_N) - l_\mu (\tilde{x}^*) + \frac{\mu }{2} |x^*-x_0|^2, \end{aligned}$$

where we have used the fact that $l_\mu (\tilde{x}^*)\le l_\mu (x ^*)$ in the second step. From the construction of $f_\mu$ we note that $|\tilde{x}^*-x_0|\le |x^*-x_0|$, which yields, when combining the previous two inequalities,

$$\begin{aligned} l(x_N)-l(x^*) \le \frac{1}{N^2} \left( \frac{5L_l}{4} |x_0-x^*|^2\right) + \frac{\mu }{2} |x^*-x_0|^2+(g(x_N)-g(x^*))^{\mathsf {\tiny {T}}}(\lambda _\mu ^*-\lambda ^*). \end{aligned}$$

The result follows from the choice of $\mu$ (i.e., $\mu =\mathcal {O}(\textrm{log}(N)^2/N^2)$), and the fact that $|\lambda _\mu ^*-\lambda ^*|\le |x^*-x_0| c_1 \mu$. $\square$

**Fig. 2:**
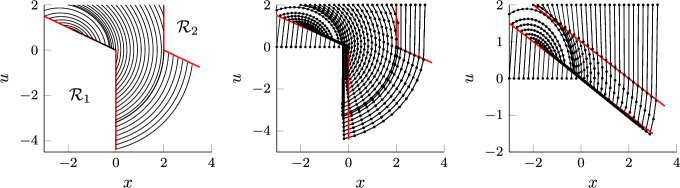
The first panel shows trajectories resulting from (7) (with parameters $\alpha =0.5, \delta =0.1, \beta =0, \epsilon =0$). The boundaries of $\mathcal {R}_1$ and $\mathcal {R}_2$ are highlighted in red. The second panel shows the results from the discretization (8) with $T_k=T=0.1$, while the third panel shows the results from the discretization (22) with $T_k=T=0.1$. An important difference between (8) and (22) lies in the fact that only violated constraints are considered in (8), whereas (22) includes all constraints. This is indicated by the red lines, which denote $\mathcal {R}_1$, $\mathcal {R}_2$ in the second panel and $\gamma _1(x,u)=0$, $\gamma _2(x,u)=0$ in the third panel

**Fig. 3:**
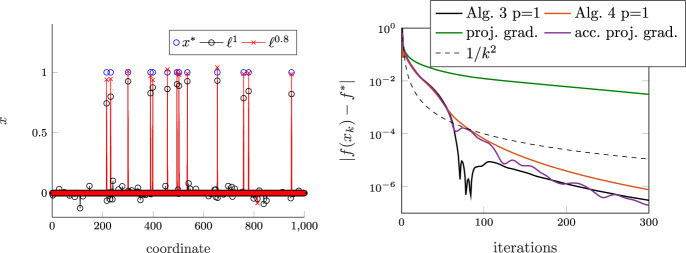
The left panel shows the solution vector of the compressed sensing problem with $\ell ^1$ and $\ell ^{0.8}$ regularization. The right panel shows the evolution of the objective function for the different methods. We note that Algorithm 3, Algorithm 4, and accelerated projected gradients converge at a similar rate, which is much faster than gradient descent. We applied the following settings for Algorithms 3 and 4: $\alpha _k=2/(k+3)$, $\delta _k=3/(2(k+3))$, $\beta _k=T(1-2\delta _k T)$ (see Table 2) with $T=1.8$ and $T=2$, respectively. Accelerated gradient descent corresponds to the algorithm from Nesterov [54, p. 78, Constant Step Scheme I]. The corresponding trajectories for Algorithms 3 and 4 for $p<1$ are similar to $p=1$ and are shown in Fig. 4

**Fig. 4:**
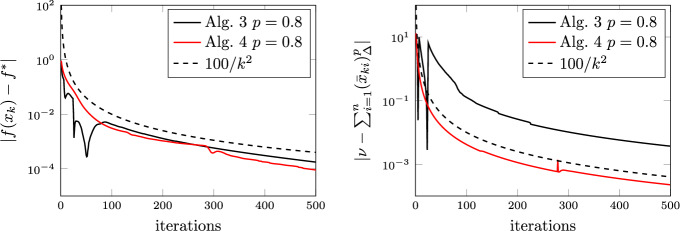
The figure shows the trajectories of Algorithms 3 and 4 applied to the compressed sensing problem with $\ell ^{0.8}$ regularization. The left panel shows the evolution of the objective function for the different methods, whereas the right panel shows the value of the constraint violation. We applied the following settings for Algorithms 3 and 4: $\alpha _k=2/(k+3)$, $\delta _k=3/(2(k+3))$, $\beta _k=T(1-2\delta _k T)$ (see Table 2) with $T=1$ and $\Delta =1e-3$

## Numerical examples

The following section is divided into two parts. The first part illustrates the dynamics of (7) and the discretization via (8) and (22) on a one-dimensional example and is intended to provide insights concerning the non-smooth dynamics, as well as the discretization. The second part applies (8) and (22) to (nonconvex) compressed sensing and large-scale sparse regression problems. As we will see, our algorithm recovers state-of-the-art performance for convex relaxations, while also handling nonconvex sparsity constraints in a seamless manner (traditional projection-based methods cannot be easily extended to this setting).

### Illustrative example

In order to plot trajectories in the phase space we choose $f(x)=(x+2)^2/2$ and $g(x)=(x,-x+2)$, where *x* is scalar. Each constraint $g_i(x)\ge 0$ and its corresponding velocity constraint $\gamma _i(x)\ge 0$ induces a region,

$$\begin{aligned} \mathcal {R}_i:=\{(x,u)\in \mathbb {R}^2~|~g_i(x)\le 0, \gamma _i(x,u)\le 0\}, \end{aligned}$$

in the phase space, $i=1,2$, where trajectories are either non-smooth or slide along the boundary of $\mathcal {R}_i$. Outside of $\mathcal {R}_i$, the trajectories follow the smooth motion (6). The first panel in Fig. 2 shows the continuous-time trajectories (7) along with $\mathcal {R}_1$ and $\mathcal {R}_2$. For a given $(x(t_0),u(t_0)^-)$ an impact happens if $g_i(x(t_0))\le 0$ and $\gamma _i(x(t_0),u(t_0))^-<0$, which ensures that $\gamma _i(x(t_0),u(t_0))^+\ge -\epsilon \gamma _i(x(t_0),u(t_0))^-$. In our example only the case $\gamma _i^+=-\epsilon \gamma _i^-$ occurs, as there are no impacts where more than one constraint participates ($\mathcal {R}_1$ and $\mathcal {R}_2$ are disjoint). The coefficient of restitution $\epsilon$ therefore determines the velocity after impact. For $\epsilon =0$ trajectories end up at the boundary of the set $\mathcal {R}_i$, whereas for $\epsilon >0$ they will leave $\mathcal {R}_i$ (in case of impact). If $g_i(x(t_0))\le 0$, $\gamma _i(x(t_0),u(t_0))^-=0$, no impact occurs, ($u(t_0)=u(t_0)^-=u(t_0)^+$), and trajectories either leave $\mathcal {R}_i$ or slide along its boundary. This depends on the contribution of the unconstrained dynamics, that is, on the vector $v_\text {uc}(t_0):=(u(t_0),-2\delta u(t_0) - \nabla f(x(t_0)+\beta u(t_0))).$ If $v_\text {uc}(t_0)$ points outwards, trajectories will leave $\mathcal {R}_i$ and follow the unconstrained motion ($\textrm{d}\lambda _i=0$). If $v_\text {uc}(t_0)$ points inwards, there will be a contribution from $\textrm{d}\lambda _i=\lambda _i(t_0) \textrm{d}t$, which ensures that trajectories slide along the boundary of $\mathcal {R}_i$.

The second panel in Fig. 2 shows the trajectories resulting from a discretization according to (8) with $T_k=T=0.1$. We can clearly see the consequences of including constraints on the velocity level: Trajectories may become infeasible, since constraints enter (8) only once they are violated. Nevertheless, even for large time steps $T_k=T$ (up to $T\approx 1.8$), trajectories converge to the unique minimizer of our problem. The third panel in Fig. 2 shows trajectories from the discretization according to (22) with $T=0.1$. We note that in (22) each constraint is permanently active (that is, $I=[n_\text {g}]$ in (2)), which explains the contrast between the trajectories in the second and third panel. In this example the dynamics (22) are more robust with respect to large time steps, and in fact, convergence can still be observed for $T=3$.

### Nonconvex compressed sensing and image reconstruction

We consider the following $\ell ^p$-regularized inverse problem:

28 $$\begin{aligned} \min _{x\in \mathbb {R}^n} \frac{1}{2} |Ax-b|^2 \quad \text {s.t.} \quad |x|_p^p\le \nu , \end{aligned}$$

where $|x|_p$ refers to the $\ell ^p$ “norm" (we explicitly allow for $0<p\le 1$). This has numerous applications in machine learning, statistics, and signal processing [see, e.g., 55]. The traditional convex approach for solving such an inverse problem is to set $p=1$ and to leverage the fact that projections onto the $\ell ^1$ ball have closed-form solutions. This yields both accelerated and non-accelerated gradient descent schemes, which in the setting of (28) are also known under the name of iterative shrinkage-thresholding (ISTA) and fast iterative shrinkage-thresholding (FISTA) [see, e.g., 56]. However, when $p<1$, projections onto the $\ell ^p$ “norm" ball no longer have closed-form solutions and it is unclear how to generalize projected gradient algorithms to this setting. Nonetheless the setting $p<1$ can be handled easily with our algorithms, as we highlight next.

In order to handle the absolute value and the *p*th power, we add slack variables, $\bar{x}\in \mathbb {R}^n$ and reformulate (28) as

29 $$\begin{aligned} \min _{(x,\bar{x})\in \mathbb {R}^{2n}} \frac{1}{2} |A x - b|^2 \quad \text {s.t.} \quad -\bar{x} \le x \le \bar{x}, \quad \sum _{i=1}^n (\bar{x}_i)_\Delta ^p \le \nu , \end{aligned}$$

where $(\cdot )_\Delta ^p: \mathbb {R} \rightarrow \mathbb {R}$ is continuously differentiable and approximates $x^p$ for $x>0$ ($x^p$, $p<1$ is nondifferentiable at the origin). The approximation $(\cdot )_\Delta ^p$ depends on the approximation parameter $\Delta >0$ and is defined as

$$\begin{aligned} (x)_\Delta ^p:={\left\{ \begin{array}{ll} x^p -\Delta ^p (1-p) &  x\ge \Delta , \\ p \Delta ^{p-1} x, &  x< \Delta . \end{array}\right. } \end{aligned}$$

For $p=1$ our approximation recovers the constraint $|x|_1\le \nu$ exactly for any $\Delta >0$. The approximation is depicted in Fig. 1 for $p=0.6$ and $\Delta =0.01$ and highlights that $x^p$ is approximated well even for modest values of $\Delta$. In the numerical examples $\Delta$ is typically set to $10^{-6}$, which yields an excellent agreement between $x^p$ and $(x)^p_\Delta$.

Despite the fact that the constraint in (29) is nonlinear and nonconvex for $p<1$, the optimization in (8) and (22) can be carried out in closed form, which yields the two algorithms Algorithms 3 and 4 stated in Appendix C.

***Nonconvex compressed sensing example:***

In the first example, each element of $A\in \mathbb {R}^{100 \times 1000}$ is sampled from a standard normal distribution. The vector *b* is set to $A x^* + n/2$, where the components of $n\in \mathbb {R}^{100}$ are sampled from a standard normal and $x^*$ is a vector that contains zeros everywhere except for 13 randomly chosen entries that are set to one. This gives rise to a challenging and ill-conditioned optimization problem that includes 1000 decision variables. The left panel in Fig. 3 compares the results computed by our algorithm for $p=1$ and $p=0.8$, whereas the right panel (solid lines) compares our approach to projected gradient descent and accelerated projected gradient descent for $p=1$. We note: (i) the quality of the reconstruction for $p=1$ is significantly worse compared to $p=0.8$ (the parameter $\nu$ was tuned with five-fold cross validation, which yielded $\nu \approx 13$ in both cases) and (ii) our algorithms decrease the objective function at a similar rate as accelerated projected gradient for $p=1$. Aside from the computation of the gradient, all algorithms have the same $\mathcal {O}(n \text {log}(n))$ complexity per iteration, which is determined by a sorting operation that is used for projecting the iterates onto the $\ell ^1$ ball or solving the minimization in (8) or (22), respectively. It is important to note that for both our algorithms the per-iteration complexity is independent of *p*. Figure 4 shows the trajectories of Algorithms 3 and 4 for $p=0.8$ and highlights a convergence rate, both in terms of function value and in terms of constraint violation, on the order of $1/k^2$ also in the nonconvex case.

***Nonconvex image reconstruction example:***

**Fig. 5:**
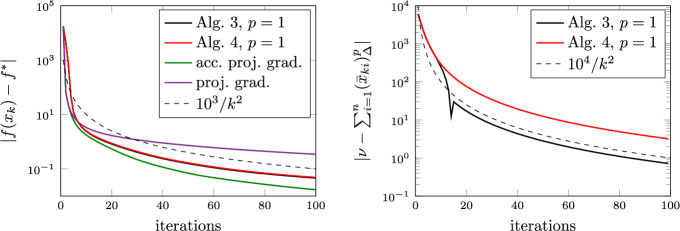
The figure on the left shows the decrease in the objective function as a function of the iterations for the different algorithms. We note that Algorithms 3, 4, and accelerated projected gradients converge at a similar rate, which is substantially faster than gradient descent. We applied the following settings for Algorithms 3 and 4: $\alpha _k=2/(k+3)$, $\delta _k=3/(2(k+3))$, $\beta _k=T(1-2\delta _k T)$ (see Table 2) with $T=1$. Accelerated gradient descent corresponds to the algorithm by Nesterov [54, p. 78, Constant Step Scheme I]. The figure on the right shows how constraint violations decrease as a function of the number of iterations. The black dashed line indicates a rate of $\mathcal {O}(1/k^2)$ as a reference. The corresponding trajectories of Algorithms 3 and 4 for $p<1$ are similar to $p=1$

**Fig. 6:**
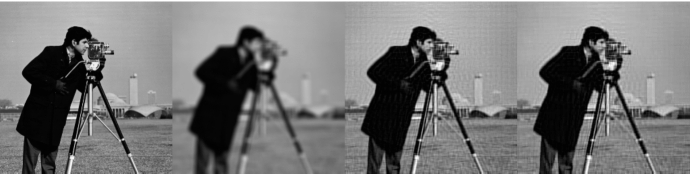
From left to right: Original image, blurred and noisy image, output after 100 iterations with accelerated gradient descent ($p=1$), output after 100 iterations with Algorithm 4 ($p=0.8$). Both the results with $p=1$ and $p=0.8$ lead to reasonable reconstructions, however, $p=0.8$ has fewer artifacts

The second example consists of an image reconstruction problem taken from Beck and Teboulle [56], where $A=RW\in \mathbb {R}^{n\times n}, n=65536$, with *R* representing a Gaussian blur operator, *W* the inverse of a three-stage Haar wavelet transform, and $\nu =6\cdot 10^{3}$. The problem given by (29) is of considerable size and includes 131,072 decision variables and 131,073 constraints. Similar to the previous example, our approach is on par with the performance of accelerated gradient descent for $p=1$, as shown in Fig. 5. However, we are also able to solve problems with $p<1$, as highlighted in Fig. 6. Figure 6 compares the resulting reconstruction of accelerated gradient descent with $p=1$ compared to our reconstruction $p=0.8$ after 100 iterations, whereby the latter has visibly fewer artifacts.

We found that the choice of the damping parameters $\delta _k$ and $\beta _k$ has a significant effect on the convergence rate. Our choice $\delta _k=3/(2(k+3)), \beta _k=1-2\delta _k$, $\alpha _k=2/(k+3)$, and $T=1$ as motivated in Table 2, is not optimized and a different schedule might lead to additional improvements.

Further details, including the derivation of Algorithms 3 and 4 from (8) and (22), are included in Appendix C.

## Conclusion

We have introduced a new type of accelerated optimization algorithm for constrained optimization problems. By imposing constraints on velocities, rather than on positions, the algorithms avoid projections or optimizations over the entire feasible set at each iteration. This not only has the potential to reduce execution time compared to Frank–Wolfe or projected gradient schemes, but more importantly, expands the range of potential applications, as constraints are not necessarily required to be convex or to have a simple structure. We have highlighted important analogies to non-smooth dynamical systems, and characterized the algorithm’s behavior in continuous and discrete time.

## Supplementary Material of Sect. 2

### Non-asymptotic rates for gradient descent

The following section contains a non-asymptotic convergence result for the gradient descent algorithm introduced in [1], which only contained an asymptotic analysis. The pseudo-code of the algorithm is listed in Algorithm 1 and requires knowledge of the smoothness constant $L_l$ of the Lagrangian. Sect. A.2 below provides results in the situation where $L_l$ is not known.

**Algorithm 1:**
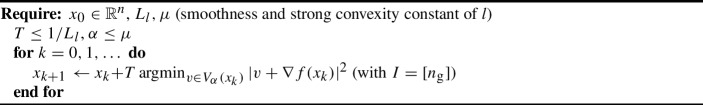
Constrained gradient descent with velocity projections

#### Theorem 7

Let the function *f* be $\mu$-strongly convex and smooth, and *g* be smooth and concave. Let $l(x):=f(x)-{\lambda ^*}^{\mathsf {\tiny {T}}}g(x)$ denote the Lagrangian with smoothness constant $L_l$, where $\lambda ^*$ denotes an optimal multiplier of (1), and let $T\le 1/L_l$, $\alpha \le \mu$. Then, the iterates of Algorithm 1 satisfy:

$$\begin{aligned} \frac{\mu }{2} |x_k-x^*|^2 ~~ \le ~~ l(x_k)-l(x^*) ~~\le ~~ (1-T \alpha )^k ~~(l(x_0)-l(x^*)), \end{aligned}$$

for all $k\ge 0$, where $x^*$ denotes the minimizer of (1). For $T=1/L_l$ and $\alpha =\mu$, the convergence is linear at rate $\mu /L_l$.

#### Proof

The proof hinges on the fact that $\alpha (x^*-x_k) \in V_\alpha (x_k)$, which follows from concavity of *g*. We define $v_k:=(x_{k+1}-x_k)/T$ to be the velocity. From the fact that $v_k=\mathop {\textrm{argmin}}_{v\in V_\alpha (x_k)} |v+\nabla f(x_k)|^2$ we conclude

$$\begin{aligned} |v_k+\nabla f(x_k)|^2/2 \le |\alpha (x^*-x_k) + \nabla f(x_k)|^2/2, \end{aligned}$$

which can be rearranged to

A1 $$\begin{aligned} |v_k|^2/2+\nabla f(x_k)^{\mathsf {\tiny {T}}}v_k&\le \alpha ^2 |x^*-x_k|^2/2 + \alpha \nabla f(x_k)^{\mathsf {\tiny {T}}}(x^*-x_k)\nonumber \\&\le (\alpha ^2-\alpha \mu ) |x^*-x_k|^2/2 + \alpha (f(x^*)-f(x_k))\nonumber \\&\le \alpha (f(x^*)-f(x_k)), \end{aligned}$$

where we have used the strong convexity of *f* in the second step and the fact that $\alpha \le \mu$ in the third step. Next we use the smoothness of *l*, which yields

$$\begin{aligned} l(x_{k+1})-l(x_k)&\le T \nabla l(x_k)^{\mathsf {\tiny {T}}}v_k + \frac{T^2 L_l}{2} |v_k|^2\\&\le T \nabla f(x_k)^{\mathsf {\tiny {T}}}v_k - T {\lambda ^*}^{\mathsf {\tiny {T}}}\nabla g(x_k)^{\mathsf {\tiny {T}}}v_k + \frac{T^2 L_l}{2} |v_k|^2\\&\le \alpha T (f(x^*)-f(x_k))- T {\lambda ^*}^{\mathsf {\tiny {T}}}\nabla g(x_k)^{\mathsf {\tiny {T}}}v_k + T \frac{TL_l - 1}{2} |v_k|^2, \end{aligned}$$

where we used inequality (A1) in the third step. We further note that by definition of $V_\alpha (x_k)$, $v_k\in V_\alpha (x_k)$ satisfies $\nabla g(x_k)^{\mathsf {\tiny {T}}}v_k \ge - \alpha g(x_k)$ and therefore $-{\lambda ^*}^{\mathsf {\tiny {T}}}\nabla g(x_k)^{\mathsf {\tiny {T}}}v_k \le - \alpha {\lambda ^*}^{\mathsf {\tiny {T}}}g(x_k)$. In addition, complementary slackness implies ${\lambda ^*}^{\mathsf {\tiny {T}}}g(x^*)=0$ and therefore

$$\begin{aligned} l(x_{k+1})-l(x_k)\le \alpha T (l(x^*)-l(x_k)) +T \frac{T L_l-1}{2} |v_k|^2. \end{aligned}$$

Subtracting and adding $l(x^*)$ on the left-hand side and using $T\le 1/L_l$ yields

$$\begin{aligned} \frac{\mu }{2} |x_k-x^*|^2 ~~ \le ~~ l(x_{k}) - l(x^*) ~~ \le ~~ (1-\alpha T)^k ~~ (l(x_0)-l(x^*)), \end{aligned}$$

which concludes the proof. $\square$

### Smoothness-agnostic rates

Theorem 7 requires knowledge of $L_l$, the smoothness constant of the Lagrangian, which requires a bound on $\lambda ^*$. This is further discussed in Appendix A, where we also add a corollary that shows that a 1/*k* rate is obtained if the smoothness constant $L_l$ is unknown. The application of Theorem 7 requires knowledge of the smoothness constant $L_l$ of *l*, which may require a bound on the optimal dual multiplier $\lambda ^*$ if *g* is nonlinear. This can be viewed as a quantitative constraint qualification, since the multiplier $\lambda ^*$ can be bounded by

$$\begin{aligned} |\lambda ^*|\le \sup _{x\in C} \frac{|W(x)^{\mathsf {\tiny {T}}}\nabla f(x)|}{\underline{\sigma }(W(x))}, \quad W(x):=(\nabla g_i(x))_{i\in I_x}, \end{aligned}$$

where $\underline{\sigma }$ denotes the minimum singular value and *W*(*x*) the matrix with columns $\nabla g_i(x)$, $i\in I_x$.

If such a quantitative constraint qualification is not available, the following corollary of Theorem 7 still applies and characterizes the convergence of Algorithm 1:

#### [Style2 Style1]Corollary 8

Let the function *f* be $\mu$-strongly convex and *L*-smooth, let *g* be concave and $L_\text {g}$-smooth, and let

$$\begin{aligned} B:=\max _{x\in C} |\nabla f(x)|^2/(2\mu ). \end{aligned}$$

Then, the iterate $x_N$ of Algorithm 1 with $T=1/(L+B L_g/\varepsilon )$, $\alpha =\mu$ satisfies $|x_N-x^*|\le \varepsilon$, where

$$\begin{aligned} N&\ge \frac{L+B L_\text {g}/\varepsilon }{\mu } \left( 2\log (1/\varepsilon ) + \log (\frac{L+B L_\text {g}/\varepsilon }{\mu }) + 2 \log (|x^*-x_0|)\right) \\  &= \mathcal {O}\left( \frac{\log (1/\varepsilon )}{\varepsilon }\right) \end{aligned}$$

and $x^*$ denotes the minimizer of

$$\begin{aligned} \min _{x\in \mathbb {R}^n} f(x) \quad \text {s.t.}\quad g(x)\ge - \varepsilon . \end{aligned}$$

#### Proof

We start by proving the following claim:

*Claim:* Let $\lambda ^*$ be an optimal multiplier of $\min _{x\in \mathbb {R}^n} f(x)~\text {s.t.}~g(x)\ge -\varepsilon$. Then $\lambda ^*$ satisfies $|\lambda ^*|_1 \le B/\varepsilon$.

*Proof of the claim:* We consider the slightly modified problem

A2 $$\begin{aligned} \min _{x\in \mathbb {R}^n, \xi \ge 0} f(x) + \bar{B} \xi /\varepsilon \quad \text {s.t.} \quad g(x)\ge - \xi , \end{aligned}$$

and denote its unique minimizer by $x^*(\bar{B}), \xi ^*(\bar{B})$ (strong convexity concludes that the minimizer is unique). In the above minimization the variable $\xi$ can be interpreted as slackness, whereby the factor $\bar{B}/\varepsilon$ penalizes the magnitude of $\xi$. The larger the factor $\bar{B}/\varepsilon$, the smaller the amount of constraint violation by the resulting $x^*(\bar{B})$. The modified problem (A2) is motivated by the fact that the corresponding Lagrange dual problem is given by

$$\begin{aligned} \max _{\lambda \ge 0, |\lambda |_1\le \bar{B}/\varepsilon } ~ \min _{x\in \mathbb {R}^n} f(x) - \lambda ^{\mathsf {\tiny {T}}}g(x), \end{aligned}$$

which implies that the minimizer $x^*(\bar{B})$ satisfies

A3 $$\begin{aligned} x^*(\bar{B})={\mathop {\textrm{argmin}}\limits _{x\in \mathbb {R}^n}} f(x) - \lambda ^*(\bar{B})^{\mathsf {\tiny {T}}}g(x), \quad \text {with}\quad |\lambda ^*(\bar{B})|_1\le \bar{B}/\varepsilon , \end{aligned}$$

and where $\lambda ^*(\bar{B})$ is an optimal dual multiplier. The maximum theorem implies that $x^*(\bar{B})$ and $\xi ^*(\bar{B})$ are continuous functions of $\bar{B}$. We will show next that for $\bar{B}=B$ the amount of constraint violation $g(x^*(B))$ is guaranteed to be below $\varepsilon$. Combined with the continuity of $\xi ^*(\bar{B})$, this implies that there exists some $B'\le B$ for which the corresponding $x^*(B')$ satisfies $x^*(B')=\mathop {\textrm{argmin}}_{x\in \mathbb {R}^n, g(x)\ge -\varepsilon } f(x)$. This in turn proves the claim in view of (A3).

In order to bound the amount of constraint violation for $\bar{B}=B$, we note that the following holds for any $x\in C$

$$\begin{aligned} f(x^*(\bar{B})) + \bar{B} \xi ^*(\bar{B})/\varepsilon \le f(x)&\le f(\hat{x}) + |\nabla f(x)|^2/(2\mu )\\&\le f(x^*(\bar{B})) +|\nabla f(x)|^2/(2\mu ), \end{aligned}$$

where strong convexity of *f* has been used in the second step and $\hat{x}$ denotes the unconstrained minimizer of *f*. Maximizing the right-hand side over $x\in C$ and rearranging terms yields

$$\begin{aligned} \xi ^*(\bar{B}) \le \varepsilon B/\bar{B}, \end{aligned}$$

which establishes that $\xi ^*(B)\le \varepsilon$ and proves the claim.

We now turn to the proof of the corollary. As a result of the claim we conclude that the minimizer $x^*$ satisfies $x^*=\mathop {\textrm{argmin}}_{x\in \mathbb {R}^n} f(x) - {\lambda ^*}^{\mathsf {\tiny {T}}}g(x)$, where the $\ell ^1$ norm of $\lambda ^*$ is bounded by $B/\varepsilon$. As a result of the choice of *T* and the parameter $\alpha$, we conclude from Theorem 7 that

$$\begin{aligned} \frac{\mu }{2} |x_N-x^*|^2 \le \left( 1-\frac{L+B L_\text {g}/\varepsilon }{\mu } \right) ^N ~~\frac{L+B L_\text {g}/\varepsilon }{2} ~~ |x_0-x^*|^2, \end{aligned}$$

where the smoothness of $f(x)-{\lambda ^*}^{\mathsf {\tiny {T}}}g(x)$ has been used to upper bound the right-hand side. The corollary follows from using the identity $1-(L+B L_\text {g}/\varepsilon )/\mu \le \exp (- (L+B L_\text {g}/\varepsilon )/\mu )$, which yields

$$\begin{aligned}&|x_N-x^*|^2 \le \frac{L+B L_\text {g}/\varepsilon }{\mu } |x_0-x^*|^2 \\&\qquad \exp \left( -2\log (1/\varepsilon ) - \log (\frac{L+B L_\text {g}/\varepsilon }{\mu }) - 2 \log (|x^*-x_0|)\right) = \varepsilon ^2 \end{aligned}$$

and proves the desired result. $\square$

## Figures and Tables

**Algorithm 2:**
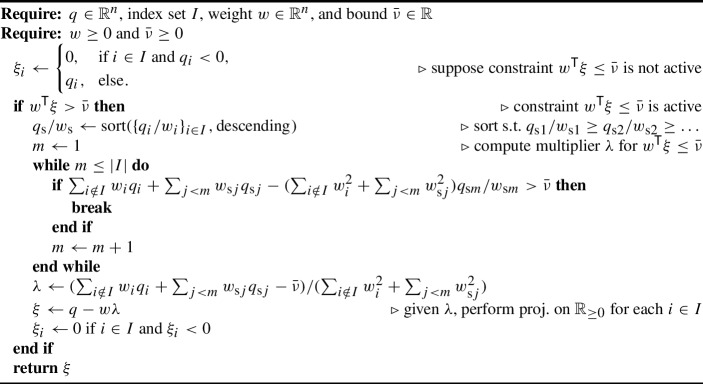
Projection onto the weighted simplex: $\mathop {\textrm{argmin}}_{\xi \in \mathbb {R}^n} |\xi -q|^2 ~~\text {s.t.}~~\xi _i\ge 0, \forall i\in I,~~w^{\mathsf {\tiny {T}}}\xi \le \bar{\nu }$

**Algorithm 3:**
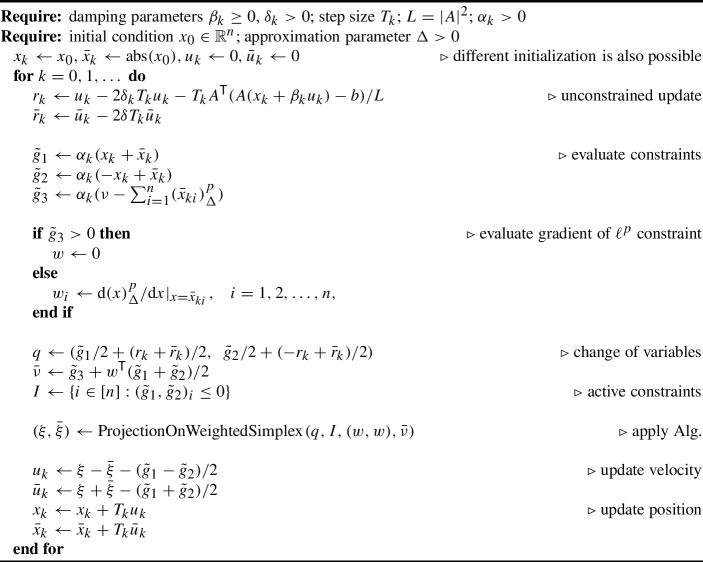
Algorithm (8) applied to (28).

**Algorithm 4:**
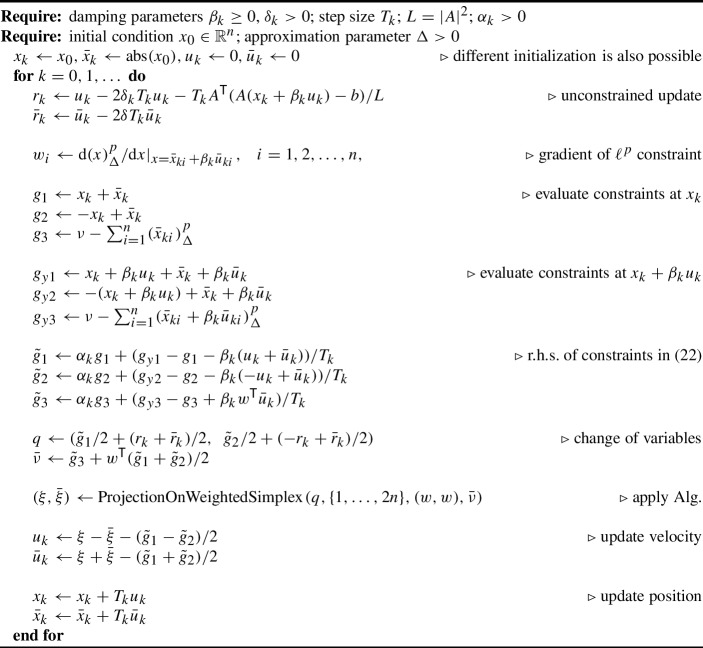
Algorithm (22) applied to (28).

## Pseudo-code of Algorithm (8)

This section lists the pseudo-code of the resulting discrete-time algorithm (8).

## Additional details on the numerical experiments

We apply (8) and (22) to (29) and start by defining the constraint function *g* as $g(x,\bar{x}):=(g_1(x,\bar{x}),g_2(x,\bar{x}))$, where $g_1(x,\bar{x}):=\bar{x}+x$ and $g_2(x,\bar{x}):=\bar{x}-x$. The decision variables $(x,\bar{x})$ will be represented with the iterates $x_k\in \mathbb {R}^n$, $\bar{x}_k\in \mathbb {R}^n$, and the corresponding velocities will be denoted by $u_k\in \mathbb {R}^n$, $\bar{u}_k\in \mathbb {R}^n$, where *k* refers to the iteration number. As a result, the optimization in (8), which is used to determine the velocities $(u_{k+1},\bar{u}_{k+1})$ can be expressed as

C4 $$\begin{aligned} (u_{k+1},\bar{u}_{k+1})=&{\mathop {\textrm{argmin}}\limits _{(v,\bar{v})\in \mathbb {R}^{2n}}} \frac{1}{2} |v-r_{k}|^2 + \frac{1}{2} |\bar{v}-\bar{r}_{k}|^2 \quad \text {s.t.} \\&\quad \bar{v}_i+v_i+\tilde{g}_{1i} \ge 0, \quad \forall i\in I_{1k},\quad \bar{v}_j-v_j +\tilde{g}_{2j}\ge 0, \quad \forall j \in I_{2k}, \nonumber \\&\quad -\sum _{i=1}^n w_l \bar{v}_l + \tilde{g}_{3}\ge 0, \nonumber \end{aligned}$$

where the last constraint only enters if $\sum _{i=1}^n (\bar{x}_{ki})_\Delta ^p \ge \nu$ holds, and where $I_{ik}$ denotes the set of constraints in $g_i$ that are active at iteration *k*, that is,

$$\begin{aligned} I_{1k}:=\{i \in [n]~|~g_{1i}(x_k,\bar{x}_k)\le 0\}, \quad I_{2k}:=\{i\in [n]~|~g_{2i}(x_k,\bar{x}_k)\le 0\}. \end{aligned}$$

The variables $\tilde{g}_1$, $\tilde{g}_2$, $\tilde{g}_3$, and *w* are defined as follows: $\tilde{g}_{1}:= \alpha g_1(x_k,\bar{x}_k)$, $\tilde{g}_2:=\alpha g_2(x_k,\bar{x}_k)$, $\tilde{g}_3:=\alpha (\nu - \sum _{i=1}^n (\bar{x}_{ki})_\Delta ^p)$, and $w_i:=\text {d} (x)^p_\Delta /\text {d}x |_{x=\bar{x}_{ki}}$. We have further set the constant $\epsilon =0$ when deriving (C4) in order to simplify the exposition. Moreover, $r_{k}\in \mathbb {R}^n$ and $\bar{r}_{k}\in \mathbb {R}^n$ are given by

$$\begin{aligned} r_{k}:=u_k-2\delta T u_k - A^{\mathsf {\tiny {T}}}(A (x_k+\beta u_k)-b) T, \qquad \bar{r}_{k}:=\bar{u}_k-2\delta T \bar{u}_k, \end{aligned}$$

where $\delta >0$ and $\beta \ge 0$ are damping parameters and $T>0$ denotes the step size. In fact, the optimization in (22) has the same form as (C4) with slightly different definitions of $\tilde{g}_1, \tilde{g}_2,$ and $\tilde{g}_3$ and all constraints being permanently active, which means, in particular, $I_{1k}=[n]$ and $I_{2k}=[n]$. In both cases, we apply the following change of variables $(v,\bar{v})\rightarrow (\xi ,\bar{\xi })$,

$$\begin{aligned} v=\xi -\bar{\xi } -\frac{\tilde{g}_1-\tilde{g}_2}{2}, \qquad \bar{v}=\xi +\bar{\xi } - \frac{\tilde{g}_1+\tilde{g}_2}{2}, \end{aligned}$$

which transforms (C4) to a projection onto a weighted simplex. The projection onto a weighted simplex has a closed-form solution and takes the form

C5 $$\begin{aligned} {\mathop {\textrm{argmin}}\limits _{(\xi ,\bar{\xi })\in \mathbb {R}^{2n}}}&\frac{1}{2} |\xi -q_1|^2+\frac{1}{2}|\bar{\xi }-q_2|^2 \quad \text {s.t.} \\&\xi _i \ge 0,~\forall i\in I_{1k}, \quad \bar{\xi }_j \ge 0,~\forall j\in I_{2k},\quad \sum _{i=1}^n w_i \xi _i + \sum _{i=1}^n w_i \bar{\xi }_i \le \bar{\nu },\nonumber \end{aligned}$$

where the last constraint only enters if $\sum _{i=1}^n (\bar{x}_{ki})_\Delta ^p \ge \nu$ and where $q_1$, $q_2$, and $\bar{\nu }$ arise from the change of variables. The weights $w_i$ are given by $\text {d}(x)_\Delta ^p/\text {d}x$ and are therefore nonnegative by construction of $(\cdot )_\Delta ^p$. The closed-form solution of (C5) is summarized in Algorithm 2 below. The solution hinges on the fact that the constraint $\sum _{i=1}^n w_i \xi _i + \sum _{i=1}^n w_i \bar{\xi }_i \le \bar{\nu }$ can be included in the objective function by means of a dual multiplier, at which point the resulting minimization corresponds to a simple projection on $\mathbb {R}_{\ge 0}$ for each $i\in I_{1k}$ and $i\in I_{2k}$. The optimal multiplier is computed by an iterative procedure that is based on a sorting operation. The worst-case complexity of Algorithm 2 is therefore $\mathcal {O}(n \text {log}(n))$.

The resulting algorithms for solving the minimization in (8) and (22) are summarized in Algorithms 3 and 4, respectively.

## Supporting Lemmas in the Proof of Theorem 3

### Lemma 9

Let the assumptions of Theorem 3 be satisfied and let $\text {dist}(x,C)$ denote the distance of $x\in \mathbb {R}^n$ to the set *C*, that is, $\text {dist}(x,C)=\min _{y\in C} |y-x|$. Then, the iterates $x_k$ are bounded, $\text {dist}(x_k,C) \rightarrow 0$, and there exists a constant $c_\text {g}>0$ and $k_0>0$ such that

$$\begin{aligned} g_i(x_k) \ge - c_\text {g} T_k, \end{aligned}$$

for all $k\ge k_0$ and all $i\in [n_\text {g}]$.

### Proof

We start by considering the first constraint, $g_1$, since $\min \{g_1(x),0\}$ has compact level sets and make the following claim:

*Claim:* There exists a constant $c_\text {g1}>0$ such that for all $k\ge 1$,

$$\begin{aligned} g_1(x_k)\ge -\frac{c_\text {g1}}{(k+c_\text {g2})^{s}}, \end{aligned}$$

where $$c_\text {g2}:= \max \{0,2s/(\alpha T_0)-1\}$$.

*Proof of the claim:* We prove the claim by induction and choose $c_\text {g1}$ sufficiently large, such that $g_1(x_1)\ge - c_\text {g1}/(1+c_\text {g2})^{s}$ (base case). We proceed to the induction step and suppose that the claim is satisfied up to iteration *k*. We now show that the claim is also satisfied for iteration $k+1$. We distinguish the following two cases.

(i) $g_1(x_k)< 0$: As a consequence of smoothness we infer that $g_1(x_{k+1}) \ge g_1(x_k) + T_k \nabla g_1(x_k)^{\mathsf {\tiny {T}}}u_{k+1} - T_k^2 L_{\text {g}1} |u_{k+1}|^2/2$, where $L_{\text {g}1}$ is the smoothness constant of $g_1$. Due to the fact that $g_1(x_k)< 0$ we have we have $1\in I_{x_k}$ and $\nabla g_1(x_k)^{\mathsf {\tiny {T}}}u_{k+1} T_k \ge -\alpha T_k g_1(x_k)$. This means that

$$\begin{aligned} g_1(x_{k+1}) \ge g_1(x_k) (1-\alpha T_k) - T_k^2 L_{\text {g}1} c_\text {u}^2/2, \end{aligned}$$

where we used the upper bound $c_\text {u}$ on $|u_{k+1}|$. As a consequence of the induction hypothesis and the choice of the stepsize $T_k=T_0/k^s$, we conclude

$$\begin{aligned} g_1(x_{k+1})&\ge -c_\text {g1}(k+c_\text {g2})^{-s} (1-\alpha T_0 k^{-s}) - T_0^2 L_{\text {g}1} c_\text {u}^2 k^{-2s}/2. \end{aligned}$$

The convexity of the function $\xi ^{-s}$ for $\xi >0$ implies

$$\begin{aligned} (k+c_\text {g2})^{-s} \le (k+1+c_\text {g2})^{-s} + s (k+c_\text {g2})^{-s-1}. \end{aligned}$$

In addition, due to the choice of $c_\text {g2}$, $-s(k+c_\text {g2})^{-1} + \alpha T_0 k^{-s}/2 \ge 0$ holds, which concludes that

$$\begin{aligned} g_1(x_{k+1})&\ge -c_\text {g1}(k+1+c_\text {g2})^{-s} + c_\text {g1} \alpha T_0 (k+c_\text {g2})^{-s} k^{-s}/2 - T_0^2 L_{\text {g}1} c_\text {u}^2 k^{-2s}/2\\&\ge -c_\text {g1}(k+1+c_\text {g2})^{-s} + c_\text {g1} \alpha T_0 (1+c_\text {g2})^{-s} k^{-2s}/2 - T_0^2 L_{\text {g}1} c_\text {u}^2 k^{-2s}/2, \end{aligned}$$

and where we have used $k^s/(k+c_\text {g2})^s \ge 1/(1+c_\text {g2})^s$ for all $k\ge 1$ in the second step. As a result, we conclude that for large enough $c_\text {g1}$ (i.e., $c_\text {g1}\ge T_0 L_{\text {g}1} c_\text {u}^2 (1+c_\text {g2})^s/\alpha$) the constraint violation $g_1(x_{k+1})$ is bounded below by

$$\begin{aligned} g_1(x_{k+1})\ge - \frac{c_\text {g1}}{(k+1+c_\text {g2})^s}, \end{aligned}$$

which proves the induction step in case (i).

(ii) $g_1(x_k) \ge 0$ and $g_1(x_{k+1}) < 0$ (the constraint $g_1$ becomes active at $k+1$): We have again $g_1(x_{k+1}) \ge \nabla g_1(x_k)^{\mathsf {\tiny {T}}}T_k u_{k+1} - T_k^2 L_{\text {g}1} c_\text {u}^2/2$ as a result of the smoothness of $g_1$ and the boundedness of $u_{k+1}$. Due to the fact that $g_1(x_k) \ge 0$ we conclude that $x_k$ is bounded ($\min \{g_1(x),0\}$ has compact level sets), which means that $|\nabla g_1(x_k)| \le \bar{c}_{\text {g1}}$ for some constant $\bar{c}_{\text {g1}}>0$. Thus, we conclude

$$\begin{aligned} g_1(x_{k+1}) \ge -T_k (\bar{c}_{\text {g1}} c_{\text {u}} + T_k L_{\text {g}1} c_{\text {u}}^2/2) \ge -\frac{T_0 (2+c_\text {g2})^s}{(k+1+c_\text {g2})^s} (\bar{c}_{\text {g1}} c_{\text {u}} + T_k L_{\text {g}1} c_{\text {u}}^2/2), \end{aligned}$$

where $1/k^s\le (2+c_\text {g2})^s/(k+1+c_\text {g2})^s$ for all $k\ge 1$ has been used in the last step.

Combining the two cases ((i) and (ii)) concludes the proof of the induction step and proves the claim. The claim implies

$$\begin{aligned} g_1(x_k)\ge -\frac{c_\text {g1}}{(k+c_\text {g2})^s}\ge - \frac{c_\text {g1}}{k^s}=-c_\text {g1}T_k /T_0, \end{aligned}$$

for all $k\ge 1$, which means that the statement of the lemma is satisfied for the first constraint. This further implies that $x_k$ is bounded, since $\min \{g_1(x),0\}$ has compact level sets. We can now apply a similar reasoning to all the remaining constraints, which establishes that $\text {dist}(x_k,C) \rightarrow 0$ and $g_i(x_k)\ge -c_\text {g} T_k$ for some constant $c_\text {g}>0$, all $i=1,2,\dots ,n_\text {g}$, and all *k* large enough. $\square$

### Lemma 10

Let $\bar{C}$ be a compact set and let *g* satisfy the Mangasarian-Fromovitz constraint qualification on $\bar{C}$. Then, there exists a constant $c_\lambda$ such that for all *x* with $x\in \bar{C}$ and $\lambda \in \mathbb {R}^{n_\text {g}}$ with $\lambda _i\ge 0$ $\forall i\in I_x$ and $\lambda _i = 0$ $\forall i\not \in I_x$,

$$\begin{aligned} |\sum _{i=1}^{n_\text {g}} \nabla g_i(x) \lambda _i| \ge c_\lambda | \lambda |. \end{aligned}$$

### Proof

The inequality is satisfied for $\lambda =0$. We therefore consider the case $\lambda \ne 0$ and without loss of generality assume that $|\lambda |=1$. We argue by contradiction and therefore assume that there are two convergent sequences, $\lambda ^j$ and $x_j$, with $|\lambda ^j|=1$, $\lambda ^j_i\ge 0$ for all $i\in I_{x_j}$, $\lambda ^j_i=0$ for all $i\not \in I_{x_j}$, and $x_j\in \bar{C}$, such that $\sum _{i=1}^{n_\text {g}} \nabla g_i(x_j)\lambda ^j_i \rightarrow 0$. Let the limit point of $x_j$ be denoted by *x* and the limit point of $\lambda ^j$ by $\lambda$. As a consequence of constraint qualification, there exists a vector $w\in \mathbb {R}^n$ such that $w^{\mathsf {\tiny {T}}}\nabla g_i(x) \lambda _i > 0$ for all $i\in I_x$. We note that $g_i(x)>0$ implies $g_i(x_j)>0$ for large enough *j*. This shows that $I_{x_j} \subset I_x$ and therefore

$$\begin{aligned} \sum _{i\in I_{x_j}} w^{\mathsf {\tiny {T}}}\nabla g_i(x) \lambda _i > c_1 \end{aligned}$$

for all large enough *j* and a small enough $c_1>0$. However, by continuity of $\nabla g$ we have

$$\begin{aligned} \sum _{i=1}^{n_\text {g}} w^{\mathsf {\tiny {T}}}\nabla g_i(x_j) \lambda _i^j&= \sum _{i \in I_{x_j}} w^{\mathsf {\tiny {T}}}\nabla g_i(x) \lambda _i + \sum _{i\in I_{x_j}} w^{\mathsf {\tiny {T}}}\nabla g_i(x) (\lambda _i^j-\lambda _i) \\&\quad + \sum _{i\in I_{x_j}} w^{\mathsf {\tiny {T}}}(\nabla g_i(x_j)-\nabla g_i(x))\lambda _i^j > c_1/2, \end{aligned}$$

for all large enough *j*, which is a contradiction. $\square$

### Lemma 11

Let the assumptions of Theorem 3 be satisfied. If $x_{k(j)} \rightarrow \bar{x} \in C$, $u_{k(j)}\rightarrow 0$, and $R_{k(j)}/T_{k(j)} \rightarrow \bar{R}$ for a subsequence $k(j), j=1,2,\dots$, then $\bar{x}$ and $\bar{R}$ satisfy

$$\begin{aligned} -\nabla f(\bar{x})+\bar{R}=0, \quad -\bar{R}\in N_{V_\alpha (\bar{x})}(0). \end{aligned}$$

### Proof

We conclude from the continuity of $g_i$ that $g_i(\bar{x})>0$ implies that $g_i(x_{k(j)})>0$ for all *j* large enough and all $i\in \{1,2,\dots ,n_\text {g}\}$. This means that $I_{x_{k(j)}}\subset I_{\bar{x}}$ for all *j* large enough. We define a slightly modified version of $V_\alpha (x)$ as follows

$$\begin{aligned} \tilde{V}_\alpha (x):=\{ u\in \mathbb {R}^n ~|~\nabla g_i(x)^{\mathsf {\tiny {T}}}u \ge -\alpha g_i(x), \quad \forall i\in I_{\bar{x}} \}, \end{aligned}$$

which ensures that $V_\alpha (x_{k(j)}) \supset \tilde{V}_\alpha (x_{k(j)})$ for large *j*. Hence, the corresponding normal cones satisfy

$$\begin{aligned} N_{V_\alpha (x_{k(j)})}(u) \subset N_{\tilde{V}_\alpha (x_{k(j)})}(u), \end{aligned}$$

for all $u\in \tilde{V}_\alpha (x_{k(j)})$, which implies, by the update rule of algorithm (8),

$$\begin{aligned} -\frac{R_{k(j)}}{T_{k(j)}} \in N_{V_\alpha (x_{k(j)})}(u_{k(j)+1}) \subset N_{\tilde{V}_\alpha (x_{k(j)})}(u_{k(j)+1}), \end{aligned}$$

where we have used the fact that the normal cone is a cone. We now show that this implies $-\bar{R} \in N_{\tilde{V}_\alpha (\bar{x})}(0)$ and argue by contradiction. This means that there exists a $\hat{u} \in \tilde{V}_\alpha (\bar{x})$ such that $-\bar{R}^{\mathsf {\tiny {T}}}\hat{u} > c_{1}$ for a small $c_{1}>0$. From constraint qualification, we infer that there exists a $w\in \mathbb {R}^n$ and an $\varepsilon >0$ such that $\hat{u} +\varepsilon w \in \tilde{V}_\alpha (x_{k(j)})$ for all *j* sufficiently large with $-\bar{R}^{\mathsf {\tiny {T}}}(\hat{u} + \varepsilon w) > c_1/2$. However, this leads to a contradiction, since

$$\begin{aligned}&0\ge -\frac{R_{k(j)}}{T_{k(j)}}^{\mathsf {\tiny {T}}}(\hat{u} + \varepsilon w - u_{k(j)+1}) \\&\quad = \underbrace{-\bar{R} ^{\mathsf {\tiny {T}}}(\hat{u}+\varepsilon w)}_{> c_1/2 > 0} + \underbrace{\bar{R}^{\mathsf {\tiny {T}}}u_{k(j)+1} - \left( \frac{R_{k(j)}}{T_{k(j)}} - \bar{R} \right) ^{\mathsf {\tiny {T}}}(\hat{u} + \varepsilon w - u_{k(j)+1})}_{\rightarrow 0}. \end{aligned}$$

This shows that $-\bar{R} \in N_{\tilde{V}_\alpha (\bar{x})}(0)$ and the desired result follows from the fact that $\tilde{V}_\alpha (\bar{x})=V_\alpha (\bar{x})$. $\square$

### Lemma 12

Let the assumptions of Theorem 3 be satisfied. Then, there exists a function $V_k: \mathbb {R}^n \times \mathbb {R}^n \rightarrow \mathbb {R}$, which is bounded below (uniformly in *k*), such that

$$\begin{aligned}&V_{k+1}(x_{k+1},u_{k+1}) - V_k(x_k,u_k) \le -c_{\text {V}1} T_k |u_k|^2 - c_{\text {V}2} |R_k|^2 + c_{\text {V}3} T_k^2 - \alpha \sum _{i\in I_{x_k}}\lambda _k^i g_i(x_k), \end{aligned}$$

for all *k* large enough, where $c_{\text {V}1}, c_{\text {V}2}, c_{\text {V}3}>0$ are constant.

### Proof

From Lemma 9 we infer that $x_k$ is bounded and therefore contained in a compact set, which we denote by $\bar{C}$. Without loss of generality we assume that *f* is analytic on $\bar{C}$ (note that by the Stone-Weierstrass theorem we can find a polynomial that approximates *f* and $\nabla f$ arbitrarily closely, see [10] for details). We can now invoke Muehlebach and Jordan [Proposition 9 of 10], which constructs the function $F_k(x,u)$ (continuous in *x* and *u*) such that

D6 $$\begin{aligned} |\tilde{H}_{k}(x_{k+1},u_{k+1})-\tilde{H}_k(\bar{x}_k,\bar{u}_k)| \le c_\text {E} T_k^4 \end{aligned}$$

for *k* sufficiently large, where

$$\begin{aligned} \tilde{H}_k(x,u):=\frac{1}{2} |u|^2 + f(x) - \frac{T_k}{2} \nabla f(x)^{\mathsf {\tiny {T}}}u + T_k^2 F_k(x,u), \end{aligned}$$

$c_\text {E}$ is constant, and $\bar{x}_k, \bar{u}_k$ are defined in (19). The function $F_k(x,u)$ is guaranteed to be bounded for $x\in \bar{C}$, $|u|\le c_\text {u}$ (uniformly in *k*). We note that (D6) implies that $\tilde{H}$ is almost conserved when applying the symplectic step in (19) (in fact, the right hand side can be replaced with $(T_k)^q$ for an arbitrarily large $q>0$). The function $\tilde{H}$ is therefore often referred to as a modified Hamiltonian and arises from the fact that the second step in (19) is a symplectic map.

We claim that the function

$$\begin{aligned} V_k(x,u):= \tilde{H}_k(x,u) + \frac{T_k d_k}{2} \nabla f(x)^{\mathsf {\tiny {T}}}u \end{aligned}$$

satisfies the desired properties, where $d_k:=1-T_k>0$ (for sufficiently large *k*). The choice $d_k=1-T_k$ is motivated by the fact that, as a result, $V_{k+1}(x,u)-V_k(x,u)$ is of order $T_k^2$ for $x_k \in \bar{C}$ and $|u_k|\le c_\text {u}$. We start by considering step 1 in (19), where we note that

$$\begin{aligned} \frac{1}{2} |\bar{u}_k|^2 - \frac{1}{2} |u_k|^2&= \frac{1}{2} (\bar{u}_k-u_k)^{\mathsf {\tiny {T}}}(\bar{u}_k+u_k)\\&=- T_k f_\text {d}^{\mathsf {\tiny {T}}}u_k + u_k^{\mathsf {\tiny {T}}}R_k + \frac{1}{2} T_k^2 |f_\text {d}|^2 - T_k f_\text {d}^{\mathsf {\tiny {T}}}R_k + \frac{1}{2} |R_k|^2\\&=- T_k f_\text {d}^{\mathsf {\tiny {T}}}u_k + u_{k+1}^{\mathsf {\tiny {T}}}R_{k} + \frac{1}{2} T_k^2 |f_\text {d}|^2 - \frac{1}{2} |R_k|^2 + T_k R_k^{\mathsf {\tiny {T}}}\nabla f(x_k), \end{aligned}$$

where we replaced $u_{k}$ by $u_{k+1}$ in the last step and omitted the arguments of $f_\text {d}(x_k,u_k)$. In addition we can bound $f_\text {d}^{\mathsf {\tiny {T}}}u_k$ and $|f_\text {d}|^2$ as follows:

$$\begin{aligned} -f_\text {d}^{\mathsf {\tiny {T}}}u_k \le - \bar{\delta } |u_k|^2, \quad |f_\text {d}|^2 \le (2\delta + \beta )^2 |u_k|^2, \end{aligned}$$

where $\bar{\delta }=2\delta$ in the convex case and $\bar{\delta }=2\delta -\beta$ in the nonconvex case. As a result, we conclude that for large enough *k*,

$$\begin{aligned} \frac{1}{2} |\bar{u}_k|^2 - \frac{1}{2} |u_k|^2 \le - \frac{T_k \bar{\delta }}{2} |u_k|^2+ u_{k+1}^{\mathsf {\tiny {T}}}R_{k} - \frac{1}{2} |R_k|^2 + T_k R_k^{\mathsf {\tiny {T}}}\nabla f(x_k). \end{aligned}$$

We further have

$$\begin{aligned} -\frac{T_k}{2} (1-d_k) \nabla f(x_k)^{\mathsf {\tiny {T}}}(\bar{u}_k-u_k)&= -\frac{T_k}{2} (1-d_k) \nabla f(x_k)^{\mathsf {\tiny {T}}}(-T_k f_\text {d} +R_k), \end{aligned}$$

which means that over step 1 in (19) the function $V_k$ changes by

$$\begin{aligned} V_{k}(\bar{x}_k,\bar{u}_k)-V_k(x_k,u_k)&\le - \frac{T_k \bar{\delta }}{2} |u_k|^2 + u_{k+1}^{\mathsf {\tiny {T}}}R_{k} + \frac{1}{2} T_k R_k^{\mathsf {\tiny {T}}}\nabla f(x_k) (1+d_k) \\&\quad - \frac{1}{2} |R_k|^2 + \frac{1}{2} T_k^2 (1-d_k) \nabla f(x_k)^{\mathsf {\tiny {T}}}f_\text {d} \\&\quad + T_k^2 \nabla _u F_k(x_k,u_k)^{\mathsf {\tiny {T}}}(-T_k f_\text {d} + R_k) + T_k^2 c_\text {F} |-T_k f_\text {d} + R_k |^2, \end{aligned}$$

where $c_\text {F}>0$ is a bound on the smoothness constant of $F_k$. We now proceed to the second step of (19), where we exploit the fact that $\tilde{H}_k$ is invariant up to terms of order $T_k^4$. We are therefore left with analyzing the term $T_k d_k \nabla f(x)^{\mathsf {\tiny {T}}}u$, which gives

$$\begin{aligned} V_k(x_{k+1},u_{k+1})\!-\!V_k(\bar{x}_k,\bar{u}_k)&\!\le \!\frac{T_k d_k}{2} (\nabla f(x_{k+1})\!-\!\nabla f(x_k))^{\mathsf {\tiny {T}}}\!u_{k+1}\\  &\quad \!-\! \frac{T_k^2 d_k}{2} |\nabla f(x_k)|^2\!+\! c_\text {E} T_k^4\\&\le \frac{T_k^2 d_k}{2} |u_{k+1}|^2 - \frac{T_k^2 d_k}{2} |\nabla f(x_k)|^2+ c_\text {E} T_k^4, \end{aligned}$$

where we used the fact that $\nabla f$ is 1-smooth in the second step. We therefore obtain

$$\begin{aligned} V_{k}(x_{k+1},u_{k+1})-V_k(x_k,u_k)&\le - \frac{T_k \bar{\delta }}{2} |u_k|^2 + u_{k+1}^{\mathsf {\tiny {T}}}R_k - \frac{d_k}{2} |R_k-T_k\nabla f(x_k)|^2 \\&\quad + \frac{T_k^2 }{2} \nabla f(x_k)^{\mathsf {\tiny {T}}}R_k + \frac{T_{k}^2 d_k}{2} |u_{k+1}|^2 + \frac{T_k^3}{2} \nabla f(x_k)^{\mathsf {\tiny {T}}}f_\text {d}\\  &\quad + T_k^2 \nabla _u F_k(x_k,u_k)^{\mathsf {\tiny {T}}}(-T_k f_\text {d} + R_k)\\&\quad + T_k^2 c_\text {F} |-T_k f_\text {d} + R_k |^2 + c_{\text {E}} T_k^4, \end{aligned}$$

where we have exploited the fact that $d_k=1- T_k$. We further note that $|\nabla _u F_k(x_k,u_k)|\le c_{\text {F}2} (|u_k|+|\nabla f(x_k)|)$ and conclude from $x_k\in \bar{C}$ and $|u_k|\le c_\text {u}$ that all variables (i.e., $\nabla f(x_k)$, $R_k$, $f_\text {d}$, $u_k$, $u_{k+1}$, and $\nabla _u F_k(x_k,u_k)$) are bounded. Hence, there exists a constant $c_V$ such that

$$\begin{aligned} V_{k}(x_{k+1},u_{k+1})-V_k(x_k,u_k) \le - \frac{T_k \bar{\delta }}{2} |u_k|^2 + u_{k+1}^{\mathsf {\tiny {T}}}R_k - \frac{d_k}{2} |R_k-T_k\nabla f(x_k)|^2 + c_V T_k^2, \end{aligned}$$

for all *k* large enough. In addition, the change of $V_{k+1}(x_{k+1},u_{k+1})-V_k(x_{k+1},u_{k+1})$ is of the order $\mathcal {O}(T_k^2)$. We further infer from the update equation for $u_{k+1}$ that

$$\begin{aligned} u_{k+1}^{\mathsf {\tiny {T}}}R_k = - \alpha \sum _{i\in I_{x_k}} \lambda _k^i g_i(x_k), \end{aligned}$$

which yields the desired result. $\square$

## Footnotes

1 This contrasts with interior-point methods, for example, which require $\mathcal {O}(\sqrt{n_\text {g}})$ Newton iterations to decrease the value of the objective by a constant factor.

2 We assume that $\dot{x}$ is of locally bounded variation, which means that on any compact interval $\dot{x}$ has countably many discontinuity points, where left and right limits exist.

3 In [45] there is a distinction between number of calls to a linear minimization oracle and number of gradient evaluations. Compared to Theorem 7, [45] requires a similar number of linear minimization calls, but fewer gradient evaluations ( [45] is accelerated in terms of gradient evaluations but not in terms of linear minimization steps).

4 We recall that the Lagrangian is given by $f(x)-{\lambda ^*}^{\mathsf {\tiny {T}}}g(x)$ with $\lambda ^*$ an optimal multiplier of (1). Hence, the smoothness constant $L_l$ is bounded by $L_f+\sum _i^{n_g} \lambda _i^* L_g^i$, where $L_g^i$ refers to the smoothness constant of $g_i$ and $L_f$ to the smoothness constant of *f*.

5 The variables $\delta$, $\beta$ may also depend on time. For ease of presentation we focus on the case where $\delta$ and $\beta$ are fixed, but also state corresponding results for time-varying parameters.

6 The $\min$ in $\gamma _i^-$ ensures $u_{k+1} \in V_\alpha (x_k)$, which is not automatically satisfied in discrete time.

7 Continuous-time rates are indeed meaningful in this context, since both the Lipschitz constant of $\nabla f$ and the constant in front of $\nabla f$ in (7) are fixed to unity. This fixes the time scale, as any reparametrization of time, i.e., $t=\tau (s)$, where $\tau :\mathbb {R}_{\ge 0} \rightarrow \mathbb {R}_{\ge 0}$ is a diffeomorphism, would alter the way $\nabla f$ enters (7). We refer the reader to Muehlebach and Jordan [9] for a more general discussion.
